# Phytochemical Profiling of *Sticta caulescens* De Not.: Green Extraction and Multiscale Chemotaxonomic Analysis

**DOI:** 10.3390/plants15111761

**Published:** 2026-06-05

**Authors:** Nicolás Cifuentes-Araya, Diego Valdivia, Mariano Walter Pertino, Daniela Marroquín-Guerra, Osvaldo Yáñez, Olimpo García-Beltrán, Alejandro Ardiles, Carlos Areche

**Affiliations:** 1 Departamento de Química, Facultad de Ciencias, Universidad de Chile, Casilla 653, Santiago 8320000, Chilediego.valdivia@ug.uchile.cl (D.V.); 2 Laboratorio de Química de Productos Naturales, Instituto de Química de Recursos Naturales, Universidad de Talca, Casilla 747, Talca 3460000, Chile; mwalter@utalca.cl; 3 Facultad de Ciencias, Ingeniería e Innovación, Universidad de Ibagué, Carrera 22 Calle 67, Ibagué 730002, Colombia; 4220211021@estudiantesunibague.edu.co; 4 Centro de Modelación Ambiental y Dinámica de Sistemas (CEMADIS), Facultad de Ingeniería y Negocios, Universidad de Las Américas, Santiago 7500975, Chile; oyanez@udla.cl; 5 Centro de Estudios e Investigación en Salud y Sociedad (EISS), Universidad Bernardo O’Higgins, General Gana 1702, Santiago 8370854, Chile; jose.garcia@unibague.edu.co; 6 Co-Laboratorio de Investigación en Bioeconomía Regional, Universidad de Ibagué, Carrera 22 Calle 67, Ibagué 730002, Colombia; 7 Departamento de Ciencias Básicas, Facultad de Ciencias, Universidad Santo Tomas, Antofagasta 1240000, Chile

**Keywords:** *Sticta caulescens*, ethyl lactate, Green extraction, microwave, non-conventional technology, chemotaxonomic analysis

## Abstract

The aim of this research was to identify the wealth of secondary metabolites in the Chilean lichen *Sticta caulescens*, applying green chemistry approaches and comparing the following two extraction methods: (a) conventional maceration with methanol, and (b) microwave-assisted extraction (MAE) using ethyl lactate as a solvent. In addition, chemoinformatic and chemotaxonomic studies were conducted on *S. caulescens* and other species of the genus *Sticta*, which have been reported in previous studies. A UHPLC/ESI-MS/MS analysis allowed for the identification of 32 metabolites obtained from maceration and 33 from MAE, considering carbohydrates, aromatic compounds, acids, depsides, depsidones, dibenzofurans, lipids, anthraquinones, and triterpenes. Maceration using methanol yielded a slightly higher extract percentage than with ethyl lactate (6.3% versus 5.0%), while MAE extracted an almost identical spectrum of metabolites using ethyl lactate,—though including one compound detected only under MAE conditions. This highlighted both the method efficiency and selectivity. This study also incorporates a comprehensive chemoinformatic and chemotaxonomic analysis of secondary metabolites across 12 *Sticta* species. A computational comparison (Morgan fingerprints, Jaccard similarity, hierarchical clustering, Murcko scaffolds) demonstrated that *S. caulescens* is one of the most chemically diverse species, closely related to *S. cordillerana*, and forming part of a major chemotaxonomic lineage, which is characterized by high scaffold richness and shared aromatic/depsidone biosynthetic pathways.

## 1. Introduction

Lichen are particular organisms that represent a unique symbiotic relationship between a fungal species, known as the mycobiont, and a photosynthetic species, referred to as the photobiont. This symbiotic association gives rise to a distinct lichen body, or thallus, which exhibits morphological, physiological, and biochemical characteristics different from those of its individual components [1,2]. The photobionts in lichen can include green algae and cyanobacteria. While lichen are often categorized as fungi due to the dominant presence of the fungal component, they differ in their nutrient acquisition mechanism [2]. The transfer of metabolites from the photobiont to the mycobiont varies depending on the type of photobiont. Eukaryotic photobionts, such as algae, transport carbohydrates in the form of sugar alcohols, whereas prokaryotic photobionts, such as cyanobacteria, transport carbohydrates in the form of glucose. Lichen rely on the photobiont for all their nutrient requirements and possess remarkable tolerance to extreme temperatures, enabling their growth to occur in diverse ecosystems [2]. The study of lichen holds significant importance due to their remarkable capacity to produce secondary metabolites, of which a substantial portion includes polyphenolic compounds derived from polyketides. Lichen are known to produce over 1200 secondary metabolites, and many of them are unique to these organisms. Notably, lichen produce a wide array of secondary metabolites, including depsides, depsidones, and dibenzofurans. These secondary metabolites exhibit diverse biological activities, such as antibacterial, antifungal, anticancer, photoprotection, and antiprotozoal activities [3,4,5]. However, our understanding of the secondary metabolites produced by certain lichen species as yet remain limited [6]. One of these species is *Sticta caulescens*, an intriguing target for the investigation of the extraction and identification techniques regarding its secondary metabolites.

Traditional extraction techniques, such as maceration, have been widely used to extract bioactive compounds from natural products. However, these methods are affected by drawbacks, such as low extraction yields, inefficiency, and the use of large volumes of potentially toxic and environmentally harmful solvents [7,8]. Consequently, there is a growing interest in the development of new extraction and separation techniques that are both effective and environmentally friendly [8,9,10]. Microwave-assisted extraction (MAE) has emerged as a promising approach for the extraction of secondary metabolites from lichen [11].

Microwave-assisted extraction utilizes microwaves, which are electromagnetic by nature, and comprise both an electric and a magnetic field, covering a frequency in the range of 300 MHz to 300 GHz. The heating mechanism in MAE is based on the interaction between the electric field and the compounds naturally present in the target material, resulting in rapid and efficient extraction [8,12]. This technique can be further classified into the following two main approaches: closed-vessel microwave-assisted extraction and microwave-assisted extraction under atmospheric pressure. Closed-vessel microwave extraction allows the process to reach higher temperatures with the simultaneous processing of multiple samples using different solvents, making it the preferred technique for the current study. In closed-vessel microwave-assisted extraction, both solubility and the dielectric constant are crucial parameters influencing extraction efficiency. Higher dielectric constants result in greater energy absorption by the solvent. MAE has been successfully applied in the extraction of various biological compounds, including the obtention of essential oils from plant leaves, glycyrrhizic acid from roots, and both ergosterol and total fatty acids from either fungal hyphae or spores [8,13].

One of the key challenges in extracting molecules from complex matrices, such as natural products, is the selection and use of proper technologies, which must enable an effective and safe extraction, improving the resultant yield with minimal negative impact on the compound’s quality [9]. Moreover, the excessive use of conventional solvents in extraction techniques poses relevant environmental and health concerns. Consequently, the current principles of green chemistry emphasize the avoidance of solvents whenever possible or their use in a manner that minimizes any negative impacts. This issue has led to the emergence of green solvents as alternatives with lower environmental impacts and improved human safety. Ethyl lactate, in particular, has gained attention as a green solvent for the extraction of secondary metabolites from lichen due to its ability to dissolve a wide range of organic compounds and its recyclability. Some studies have provided a comparison of the relevant parameters between conventional solvents, such as n-hexane, toluene, dichloromethane, and the aforementioned green solvents [14,15,16].

In this study, we aimed to explore the application of microwave-assisted extraction, specifically the closed-vessel approach, for the extraction of secondary metabolites from *Sticta caulescens* lichen. Further, we investigated the use of ethyl lactate as a green solvent for the extraction process. By employing this green extraction technique, we aimed to enhance our understanding of the secondary metabolites present in *S. caulescens*, and to contribute to the development of green practices related to natural product extraction. In addition, we compared this technique with the traditional extraction method of maceration with MeOH as a solvent. A chemoinformatic analysis provided insight into the metabolic diversity and chemotaxonomic relationships of lichen of the genus *Sticta*, including the revealed chemical patterns that are consistent with known taxonomic groupings within the genus, and showing the quantitative frameworks that complement classical taxonomy. Chemotaxonomic analyses based on molecular fingerprints, scaffold identification, and similarity clustering allowed us to explore the biosynthetic patterns. These would otherwise be difficult to resolve through morphology alone. By integrating high-resolution metabolomic profiles with computational analyses across multiple *Sticta* species, this work not only clarifies the chemical uniqueness and phylogenetic placement of *S. caulescens* but provides broader insight into metabolic diversification within the genus studied.

## 2. Results and Discussions

### 2.1. Extraction Using Conventional and Non-Conventional Techniques

The powdered samples (1 g) of *S. caulescens* were extracted with MeOH and ethyl lactate, using conventional and non-conventional techniques, respectively. Maceration was used in the first case, and microwave in the second case. Extractions with methanol, and ethyl lactate (MAE) yielded 6.3 ± 0.3%, and 5.0 ± 0.3%, respectively. Maceration is a simple technique, low-cost, time-consuming, and easy to perform, but it consumes a significant amount of solvent, and typically yields only moderate results. On the other hand, microwave-assisted extraction achieves higher yields of compounds, reduces both the processing time and solvent use, and is more energy-efficient. However, it requires modern equipment and careful handling due to the high temperatures and pressures applied. In general, maceration is a suitable option when simplicity and low cost are desired, whereas microwave extraction is preferable when speed, greater efficiency, and higher yields are required [10,16]. Our findings indicate that methanol performs better than ethyl lactate as the extraction solvent based on its obtained yield.

### 2.2. Metabolomic Profiling Using UHPLC-ESI-MS/MS

The tentative identification of lichen substances is the main bottleneck on the structural elucidation based on the MS/MS data, making the process a huge time-consuming task. As a strategy, some software were explored, as well as databases including NIST, METLIN, MassBank, CFM-ID, MS-DIAL, and ACD LAB. In this sense, the chemical structures of unknown compounds were tentatively proposed, based on MS/MS fragmentation strategies of the main small fragments, as well as considering the biosynthetic pathway of the compounds. During our study, both the ethyl lactate and methanolic extract profiling were chosen for a comparative purpose, as shown in Table 1. All the identified compounds were revised and checked by comparing them with the previously published LC/MS/MS data [17]. The methanolic extract obtained under maceration gave thirty-two compounds, while, for the use of ethyl extract assisted by microwave, thirty-three compounds were detected.

*Carbohydrates*

Peak 1 was tentatively identified as manitol, with a molecular ion at *m*/*z* 181.0710. It was found in both extracts [17].

*Acids*

Peaks 3 and 4 were assigned to the metabolites gluconic acid and citric acid, respectively. The two compounds were found in both extracts [17].

*Depsides*

Peak 11 was tentatively identified as nor-8′-methylconstictic acid, with *m*/*z* 447.0921 [17]. Peaks 17 and 23 were assigned to 4-O-demethylbarbatic acid and to a fumarprotocetraric acid derivative, with *m/z* 345.0985 and 311.0561, respectively. The identified 8′-methylconstictic acid displayed a daughter ion at *m/z* 209.0510 (breaking of an ester bond). These compounds were found in both the methanolic and ethyl lactate extracts [17].

*Aromatics*

Peaks 5 and 32 were assigned to 2-ethylisophthalic acid and methyl orsellinate, with *m*/*z* 193.0511 and 181.0516, respectively [17]. Peaks 6, 7, 20, and 24 were tentatively identified as orsellinic acid, trihydroxy benzaldehyde, 2-ethylisophthalic acid I, and 2,4-dihydroxy benzaldehyde I, respectively [17]. Peaks 8, 9, and 12 were identified tentatively as 2,4-dihydroxy benzaldehyde, 5,7-dihydroxy-6-methylphthalide, and 2-hydroxyisophthalic acid, respectively. The identified 5,7-dihydroxy-6-methylphthalide showed a daughter ion at *m*/*z* 135.0497, and 2-hydroxyisophthalic acid displayed a daughter ion at *m*/*z* 137.0288 (loss of a carboxylic acid as CO_2_). All these compounds were found in both extracts [17].

*Anthraquinones*

Peaks 13 and 22 were assigned to parietin and pentahydroxy-3-methylanthraquinone, with *m*/*z* 283.0613 and 301.0359, respectively [17]. Peak 29 was tentatively identified as parietin I, with *m*/*z* 283.0620, and with a daughter ion at *m*/*z* 149.0248 [17]. These compounds were found in both the methanol and ethyl lactate extracts.

*Lipids*

Ten lipids were tentatively identified in both organic extracts through peaks 14, 15, 21, 25, 26, 28, and 34–37 [11,17].

*Dibenzofurans*

Peak 16 was tentatively identified as usnic acid, with a molecular ion at *m*/*z* 343.0825, and it was found in both extracts [17].

*Depsidones*

Peak 18 was identified as didechlorolecideoidin displaying a molecular ion at *m*/*z* 329.0672 and a daughter ion at *m*/*z* 285.0855 [17]. Peak 19 was assigned to hypostictic acid isomer with *m*/*z* 371.0789, with daughter ions at *m*/*z* 327.0987 (loss of an ester group as CO_2_) and 179.0391 (breaking of an ester bond). These compounds were found in both the methanolic and ethyl lactate extracts.

*Pulvinic acid and derivatives*

Peak 33 was tentatively identified as a pulvinic acid derivative, with a molecular ion at *m*/*z* 323.0550. It was found in the ethyl lactate extract and not in the methanolic extract [17].

*Triterpenes*

During our study, three triterpenoids were tentatively identified, including retigeric acid B (peak 30) and retigeric acid derivatives (peaks 27 and 31), with molecular ions at *m*/*z* 501.3228, 555.2969 and 487.3440, respectively [17]. These three triterpenoids were detected in both extracts.

The use of ethyl lactate as a versatile solvent for the extraction and separation of a wide range of secondary metabolites has increased lately. Some results have been reported showing the potential use of this solvent for the extraction of carotenoids and its congeners, tocopherol, sclareol, quercetin, rutin, caffeine, amino acids, and phenolics from lichen [18,19,20,21,22,23,24,25,26]. In the case of lichen compounds, Sepulveda et al. (2023) [27] reported their green extraction assisted by ultrasounds from *Hypotrachyna cirrhata* lichen using ethyl lactate as solvent, and its comparison with maceration in methanol. They showed that the ultrasound-assisted extraction using ethyl lactate was comparable to the maceration-assisted extraction regarding its phytochemical profile. Moreover, the LC/MS/MS analysis provided 70 compounds for the ethyl lactate extract and 69 metabolites for the MeOH extract, both obtained from the lichen *Hypotrachyna cirrhata*. In this sense, the in vitro antioxidant assays (DPPH, FRAP, ABTS and TPC) performed on the ethyl lactate extract showed a higher activity than that realized on the methanol extract, concluding that the green extract’s antioxidant activity was significantly higher [27]. On the other hands, Castañeta et al. (2023) [28] informed that the LC/MS/MS data provided 20 compounds for an ethyl lactate extract, 4 for a limonene extract, and 14 metabolites for an MeOH extract, all of them obtained from the lichen *U. cornuta*. Regarding the in vitro antioxidant assays, in that study, the ethyl lactate extract (562.61 µM/g of dry lichen) showed a higher activity than the methanol extract (288.15 ± 0.05 µM/g of dry lichen), based on an ORAC assay. The findings made by Castañeta et al. (2023) [28] showed that the extraction of metabolites using green solvents combined with MAE is a better alternative than the use of toxic organic solvents, such as methanol. When comparing those reports with our study, the trend observed is the same, based on the observed phytochemical profile. In our study, both extracts had a similar profile based on the UHPLC/ESI/MS/MS analysis. In the case of maceration with methanol, untargeted metabolomics tentatively identified 32 compounds obtained from the methanolic extract, while, from MAE with ethyl lactate solvent, 33 metabolites were identified. The main difference was in peak 33, assigned to the pulvinic acid derivative. These results of lichen substance profiles confirmed the comparative efficiency of ethyl lactate with MeOH for recovering the range of compounds from *S. caulescens* extracts. The comparable extraction efficiency of ethyl lactate relative to MeOH arises from its favorable hydrogen-bonding capacity, amphiphilic profile, and a dielectric constant that enables it to solvate lichen substances. This was due to the presence of phenolics, aldehydes, and carboxylic acids in the *S. caulescens* extracts, relating hydrogen bonding to efficient extraction. These factors collectively explain the broadly comparable extraction performance of ethyl lactate solvent observed in this study.

The use of unconventional techniques along with green solvents could represent a new way for the extraction and isolation of secondary metabolites from plants, lichen, and other organisms. The benefits of combining both techniques would result in more safety, efficiency, and greenness in many chemical processes. This way, the latter could be better applied in drug discovery, development of new materials, and environmental sciences.

Finally, ethyl lactate has established itself as one of the most promising and environmentally friendly solvents for the extraction and isolation of secondary metabolites from lichen. This solvent is produced through the fermentation of renewable carbohydrates and subsequently by esterifying lactic acid with ethanol, making it a fully biological and biodegradable product [29]. Solvents traditionally used in lichen research, such as methanol, hexane, and chloroform, inevitably contribute to environmental pollution and pose severe risks to human health, making their replacement an urgent priority, and in line with the twelve principles of green chemistry [27]. The advantages of ethyl lactate are particularly relevant when applied to the extraction and isolation of secondary metabolites from lichen, a group of organisms characterized by structurally diverse and often hydrophobic compounds including lipids, organic acids, depsides, depsidones, and dibenzofurans [11,27,28].

### 2.3. Chemoinformatic and Chemotaxonomic Analysis of the Sticta genus Species

To elucidate the chemical relationships within the genus *Sticta*, 11 congeneric species (*S. andina, S. hypoglabra, S. cordillerana, S. gyalocarpa, S. leucoblepharis, S. parahumboldtii, S. impressula, S. ocaniesnsis, S. pseudosylvatica, S. luteocyphelata,* and *S. lineariloba*) were compared with *S. caulescens* through a comprehensive chemoinformatic analysis. A binary presence/absence matrix was constructed to systematically evaluate the distribution of secondary metabolites across all species. Chemical structures were encoded using the Simplified Molecular Input Line Entry System (SMILES) notation, enabling the computational assessment of molecular fingerprints and structural scaffolds. Chemotaxonomic relationships were established through hierarchical clustering based on Tanimoto similarity indices derived from Morgan fingerprints, thereby revealing species-specific metabolic profiles and shared biosynthetic pathways. A Murcko scaffold analysis identified the conserved structural motifs across the genus, with benzene and depside/depsidone cores representing the most prevalent chemotypes. This integrative approach combining metabolomic profiling with chemoinformatic tools provides molecular evidence for chemotaxonomic grouping and metabolic differentiation within *Sticta* lichen. It should be noted that chemotaxonomic clustering based on secondary metabolite profiles reflects chemical affinity and may or not fully correspond to any phylogenetic relationships inferred from the sequence data. The patterns reported here should therefore be interpreted as chemically-based hypotheses that would benefit from corroboration with molecular phylogenetic analyses.

The comparative metabolomic analysis revealed substantial variation in secondary metabolite richness across the twelve *Sticta* species examined, ranging from 14 compounds (*S. leucoblepharis*, *S. pseudosylvatica*, *S. luteocyphelata*) to 32 compounds (*S. cordillerana*, *S. caulescens*), representing a 2.3-fold difference in chemical complexity. A statistical analysis of compound distribution demonstrated that each metabolite occurred in an average of 2.28 ± 2.18 species, with highly skewed distribution patterns evidenced by the median and first quartile values of 1.0 species. This indicates that the majority of secondary metabolites exhibited taxonomic specificity, with 75% of all compounds occurring in two or fewer species. Only a small fraction of the identified metabolites showed a broad distribution, with the maximum occurrence reaching 11 of 12 species. These findings suggest that most *Sticta* species possess distinctive chemical fingerprints dominated by species-specific or clade-restricted metabolites, while a conserved core of widely distributed compounds likely represents essential biosynthetic pathways.

**Figure 1:**
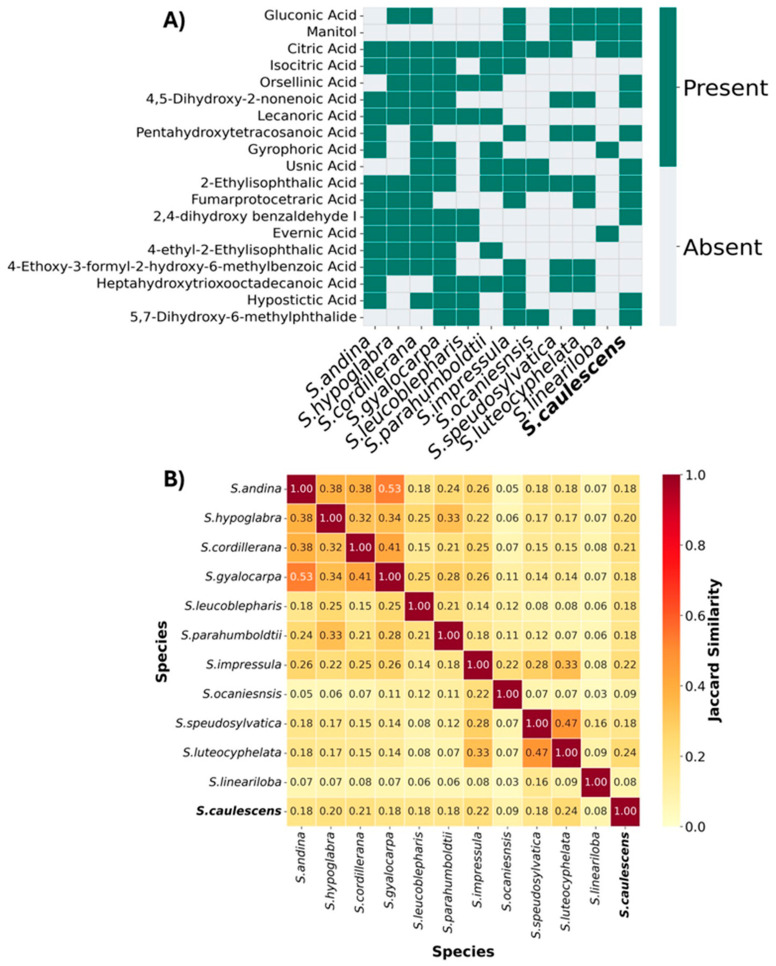
(**A**) Absence and presence of molecules in the 12 different species of *Sticta* lichen is shown. A threshold of over 40% was considered for a more considerable graph size (for the complete graph, see the Supplementary Materials, Figure S1). (**B**) Heatmap of Jaccard similarity matrix, where 1 is very similar and 0 is very dissimilar.

Figure 1A reveals the significant patterns in the distribution of secondary metabolites across the 12 lichen species of the genus *Sticta*. Chemical diversity demonstrates clear interspecific heterogeneity, suggesting a differentiated metabolic specialization across the genus. These patterns may reflect underlying evolutionary processes, although formal evolutionary inferences would require an integration with sequence-based phylogenetic data. Citric acid emerges as the most ubiquitous metabolite, present in virtually all the analyzed species, and indicating a fundamental role in the primary metabolism or basal chemical defenses. By contrast, specialized compounds, such as gyrophoric acid and evernic acid, exhibited restricted distributions, possibly related to specific ecological niches or particular selective pressures. The species *S. cordillerana*, *S. gyalocarpa*, and *S. andina* presented more complex metabolic profiles, suggesting greater biosynthetic capacity or chemical diversification. Notably, compounds like lecanoric acid and pentahydroxytetracosanoic acid showed an intermediate presence, suggesting conditional regulation or environmentally dependent expression. The systematic absence of certain metabolites in species like *S. luteocyphelata* could reflect the loss of biosynthetic pathways or alternative metabolic specializations. This chemotaxonomic analysis provided valuable information regarding chemical classification and identification of species-specific biomarkers, complementing the available phylogenetic frameworks. It is important to note that metabolite-based groupings represent chemical hypotheses, and should be integrated with molecular sequence data to generate formal phylogenetic conclusions [30,31] and to understand defensive strategies in these symbiotic organisms. The distinct metabolite signatures may also have implications for ecological interactions, lichen-associated microbiota, and adaptation to diverse environmental conditions across geographical distribution ranges.

The Jaccard similarity matrix (Figure 1B) revealed that *S. caulescens* exhibited moderate-to-low chemical similarity with other *Sticta* species, with coefficients ranging from 0.08 to 0.24. The highest metabolic affinity was observed with *S. luteocyphelata* (J = 0.24), followed by *S. impressula* (J = 0.22) and *S. cordillerana* (J = 0.20), suggesting these species share approximately 20–24% of their secondary metabolite repertoires with *S. caulescens*. Conversely, *S. lineariloba* exhibited the lowest similarity (J = 0.08), indicating substantial metabolic divergence and suggesting minimal overlap in biosynthetic pathways. Notably, all Jaccard coefficients remained below 0.25, demonstrating that *S. caulescens* maintained a predominantly unique chemical profile with over 75% species-specific or distinct metabolites relative to each congener examined. The intermediate similarity values with *S. hypoglabra* (J = 0.20), *S. andina* (J = 0.18), and *S. gyalocarpa* (J = 0.18) reflected moderate metabolic overlap, potentially representing conserved core biosynthetic pathways within the genus. The remarkably low similarity with *S. ocaniesnsis* (J = 0.09) further emphasized the metabolic heterogeneity within the *Sticta* genus. These findings underscore the chemical distinctiveness of *S. caulescens* and support its recognition as a metabolically discrete taxon within the genus, with chemotaxonomic relationships most closely aligned with *S. luteocyphelata* and *S. impressula*.

**Figure 2:**
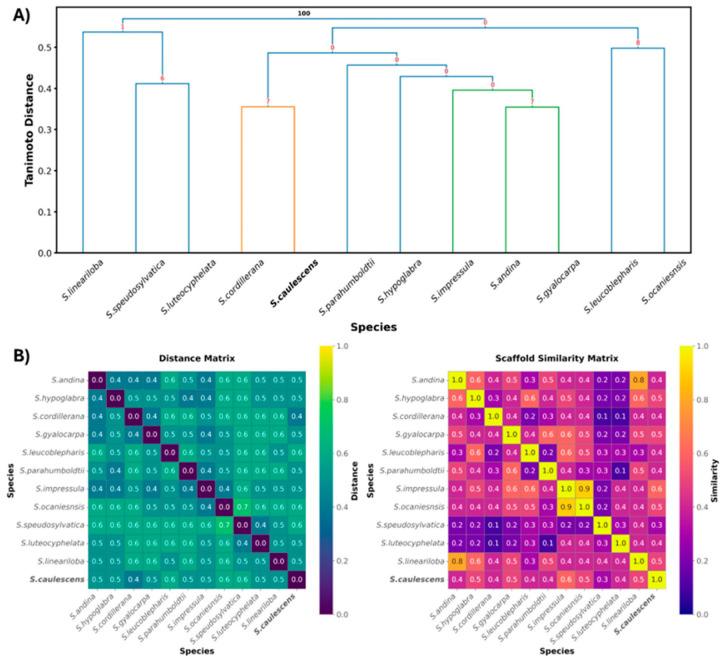
The comparative molecular dendrogram (**A**) illustrates chemotaxonomic relationships among the *Sticta* species based on secondary metabolite profiles. These groupings reflect chemical similarity rather than formal phylogenetic inference, and should be interpreted as metabolite-based hypotheses pending validation against sequence-based phylogenies, where a distance of zero indicates near-identical chemical profiles and a value approaching one reflects substantial divergence in metabolite composition. Panel (**B**) presents the pairwise distance matrix, quantitatively revealing the levels of metabolic similarity and disparity within the genus. Additionally, the scaffold similarity matrix highlights the degree of overlap for ring-containing structural motifs among species, identifying the conserved aromatic cores and chemotaxonomic clusters. Together, these analyses offer integrated molecular evidence for evolutionary differentiation and structural convergence in *Sticta* lichen. The numbers in red are identifiers of the internal nodes of the dendrogram that mark the successive steps of the hierarchical grouping process based on the Tanimoto distance between metabolite profiles.

The hierarchical clustering analysis (Figure 2) of the twelve *Sticta* species revealed profound metabolic heterogeneity across the genus, with pairwise distances spanning from 0.35 to 0.65 (mean = 0.52 ± 0.07) [17,32]. The dendrogram identified three principal chemotaxonomic lineages distinguished by distinct secondary metabolite profiles. The first major clade, comprising *S. andina*, *S. gyalocarpa*, and *S. impressula*, exhibited the highest intermolecular similarity (distances 0.35–0.39). This suggested conserved biosynthetic pathways and chemical affinity that may indicate shared evolutionary origins. Nevertheless, this interpretation should be regarded as tentative in the absence of molecular phylogenetic data [33]. This cluster subsequently incorporated *S. hypoglabra* and *S. parahumboldtii* at moderate distances (0.429–0.457), forming an expanded metabolically cohesive group. Notably, *S. ocaniesnsis* and *S. pseudosylvatica* constituted the most chemically divergent pair (distance = 0.659), indicating only 34% metabolic overlap and substantial functional differentiation in secondary metabolism. A peripheral chemotype cluster united *S. leucoblepharis*, *S. ocaniesnsis*, and *S. lineariloba*, which displayed greater metabolic isolation from the core dendrogram (fusion distance = 0.56). The *S. pseudosylvatica*–*S. luteocyphelata* subclade (distance = 0.41) represented an intermediate metabolic signature. A statistical analysis confirmed moderate average divergence across the genus (mean distance = 0.52) with relatively low variance (σ = 0.07), suggesting a gradual metabolic diversification rather than abrupt biochemical innovation [34]. Within this chemotaxonomic framework, *S. caulescens* occupies a distinctive position, clustering most closely with *S. cordillerana* at a remarkably low distance of 0.35, the second-smallest pairwise distance observed across all species comparisons. This tight phylochemical association indicates that *S. caulescens* and *S. cordillerana* share approximately 64.5% of their metabolic profiles, reflecting convergent or shared biosynthetic capacities. The *S. cordillerana*–*S. caulescens* dyad merged with the larger *S. andina*-dominated clade at a distance 0.48, positioning *S. caulescens* within the primary metabolic lineage of the genus, while maintaining a sufficient chemical distinctiveness to warrant taxonomic recognition as a discrete entity [17].

In the fingerprint-based chemotaxonomic dendrogram, *S. caulescens* clusters with *S. cordillerana* at a Tanimoto distance of 0.355 (similarity = 0.645), which is the second smallest pairwise distance among all species. However, these two species have in common only 12 of the 51 reported metabolites (Jaccard coefficient = 0.2353), indicating that this grouping is driven by overlapping molecular substructures rather than by a large number of identical compounds. Consistently, the bootstrap support for this node is low (6.8%), and we therefore interpret this relationship as tentative and highly sensitive to the current incompleteness and publication bias of the chemotaxonomic dataset.

It is necessary to acknowledge the existence of certain limitations of this chemotaxonomic analysis. First, the dataset comprises only 12 *Sticta* species and 109 reported metabolites, which restricts the statistical power of the clustering. Second, the metabolite profiles are affected by publication bias, as more extensively studied species are likely overrepresented. Third, the Tanimoto distance between consensus Morgan fingerprints reflects similarity at the level of shared molecular fragments. Therefore, it systematically exceeds the Jaccard coefficient calculated from binary presence/absence data. In particular, the clustering of *S. caulescens* with *S. cordillerana* at a Tanimoto distance of 0.355 is supported by relatively few shared compounds (Jaccard = 0.2353), and exhibits low bootstrap support (6.8%). Thus, this relationship should be regarded as provisional until more comprehensive metabolomic data become available.

A fingerprint analysis of the molecular substructures revealed substantial variation in chemical diversity across the twelve *Sticta* species examined, with active bit percentages ranging from 16.11% to 31.05%. The majority of the species exhibited moderate chemical diversity (16–24% active bits), including *S. leucoblepharis* (16.11%), *S. luteocyphelata* (16.41%), *S. parahumboldtii* (16.99%), *S. pseudosylvatica* (17.87%), *S. lineariloba* (18.65%), *S. ocaniesnsis* (20.12%), *S. hypoglabra* (21.48%), *S. impressula* (21.88%), and *S. andina* and *S. gyalocarpa* (both 23.54%). These intermediate values suggest balanced metabolic repertoires with moderate structural complexity. By striking contrast, two species demonstrated a significantly elevated chemical diversity, namely *S. caulescens* (29.49%) and *S. cordillerana* (31.05%), reflecting approximately 1.5–1.9-fold greater substructural complexity when compared to the least diverse species. This pattern indicates that *S. cordillerana* and *S. caulescens* possess the most elaborated secondary metabolite architectures within the genus, potentially driven by adaptation to diverse ecological niches or expanded biosynthetic gene clusters. Notably, *S. caulescens* ranks as the second most chemically diverse species, with only *S. cordillerana* surpassing it by a narrow margin (1.56 percentage points). This alignment corroborates the close phylochemical relationship between these two species observed in hierarchical clustering (distance = 0.355). This suggests that their shared metabolic complexity represents a conserved trait within this chemotaxonomic lineage.

**Figure 3:**
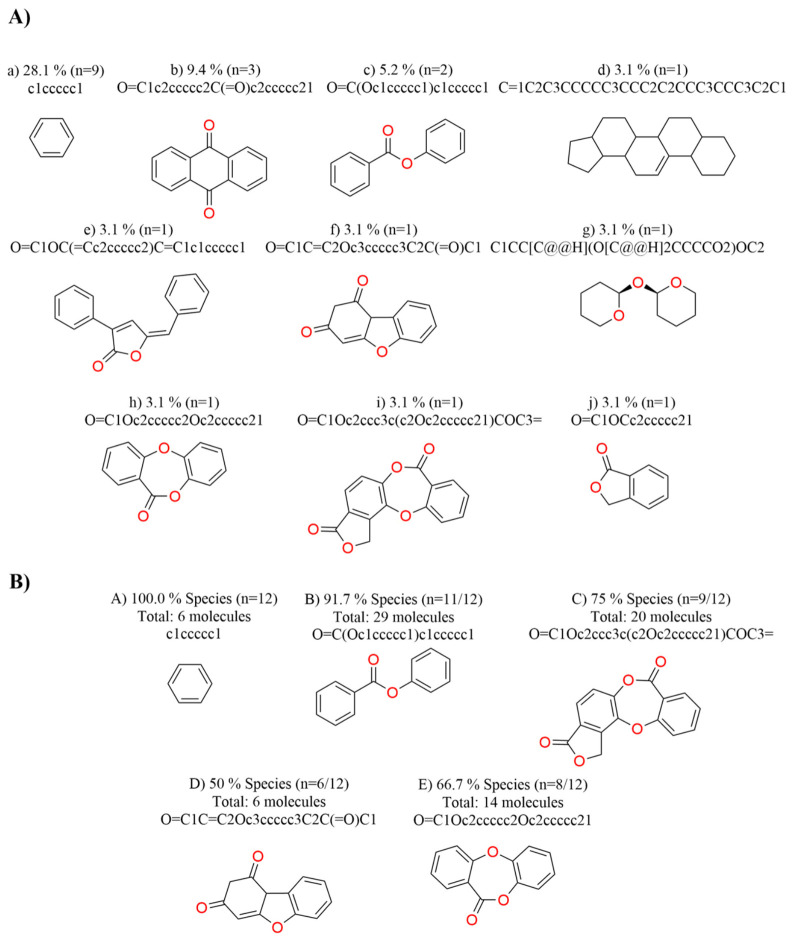
Predominant ring-containing scaffolds identified in (**A**) *S. caulescens* and (**B**) *Sticta* species, including the frequency of each scaffold (as a percentage).

A Murcko scaffold analysis revealed a pronounced variation in core structural diversity across the twelve *Sticta* species, ranging from 4 to 12 unique scaffolds per species. The species *S. cordillerana* exhibited the highest scaffold richness (12 scaffolds from 32 compounds, 37.5% unique), followed closely by *S. caulescens* (10 scaffolds from 32 compounds, 31.3% unique), indicating an exceptional structural heterogeneity within these taxa. Intermediate diversity was observed in *S. gyalocarpa* (8/26), *S. andina* (7/26), *S. ocaniesnsis* (7/15), and *S. lineariloba* (7/22), while *S. pseudosylvatica* and *S. luteocyphelata* displayed minimal scaffold diversity (4/14 each, 28.6%) (see the Supplementary Materials, Figures S2–S12). The scaffold repertoire of *S. caulescens* (Figure 3A) encompassed diverse chemotypes including simple aromatic cores (a-benzene, c-phenyl benzoate), oxidized polycyclic systems (b-anthraquinone, f-dibenzofuran-dione), complex depsidone frameworks (h-dibenzo[b,e]dioxepin-11-one, i-isobenzofuro-benzodioxepin-dione), lactone motifs (j-phthalide, e-benzylidene-phenylfuranone), terpenoid-derived polycycles (d-cyclopenta[a]chrysene), and glycosidic scaffolds (dioxane linkages). This structural breadth reflects a multifaceted biosynthetic capacity, encompassing aromatic polyketide, shikimate-derived, and terpene-mevalonate pathways. Notably, *S. caulescens* shares the benzene and phenyl benzoate scaffolds with most congeners, whereas depsidone and dibenzofuran cores represent chemotaxonomic markers characteristic of *Sticta* lichen. The presence of ten distinct scaffolds positions *S. caulescens* as one of the most structurally versatile species within the genus, second only to *S. cordillerana*, which is consistent with their phylochemical proximity and high metabolic complexity.

The analysis of the five most prevalent scaffolds revealed conserved biosynthetic frameworks across the genus *Sticta* (Figure 3B), with benzene (scaffold a) representing a universal structural motif being present in all twelve species examined (100% occurrence, 51 compounds, 20.48% of total metabolites) [17]. This ubiquitous aromatic core underlies the shikimate-derived biosynthetic pathways that are fundamental to lichen secondary metabolism [35,36]. Phenyl benzoate (scaffold b) exhibited a near-universal distribution across 11 of 12 species (91.7%, 29 compounds, 11.65%), representing the characteristic depside linkage formed through esterification of aromatic carboxylic acids, a hallmark of *Sticta* chemistry [37]. The depsidone-type scaffold 7H-isobenzofuro [4,5-b]benzodioxepin-3,7(1H)-dione (scaffold c) was detected in nine species (75.0%, 20 compounds, 8.03%), including *S. caulescens*, reflecting a widespread capacity for oxidative cyclization of depside precursors into complex tricyclic frameworks [38]. The structurally related dibenzo[b,e]dioxepin-11-one (scaffold d) occurred in eight species (66.7%, 14 compounds, 5.62%), whereas the anthraquinone-derived dibenzofuran-1,3-dione (scaffold e) was restricted to six species (50.0%, 6 compounds, 2.41%), demonstrating a progressive narrowing of biosynthetic distribution for more specialized oxidative transformations [32,39]. Notably, *S. caulescens* harbors all five conserved scaffolds, positioning it as a metabolically representative species that retains both ancestral core pathways (benzene, phenyl benzoate) and derived specialized frameworks (depsidones, dibenzofurans). This is consistent with its high scaffold diversity and central phylochemical placement within the genus.

## 3. Materials and Methods

### 3.1. Plant Material

Specimens were collected in 2023 at Longaví, Maule Region, Chile, and identified as *Sticta caulescens* by Dr. Reinaldo Vargas from Universidad Metropolitana de Ciencias de la Educacion (UMCE), Santiago, Chile. A voucher specimen (Nº STC-09112023) was deposited at the Herbarium of the UMCE.

### 3.2. Chemicals Reagents

Ethyl lactate and MeOH were purchased from Sigma Aldrich (Saint Louis, Mo, USA).

### 3.3. Maceration Extraction

The ground dried sample of *S. caulescens* (1.0 g) was placed into 10 mL of analytical-grade methanol at room temperature under constant stirring (200 rpm), and extracted for 72 h. This procedure was performed twice per week. After centrifugation (9000× *g*, 30 min), the supernatant was filtered and concentrated in vacuum conditions to yield 62.2 ± 2.5 mg of a dark extract (6.3 ± 0.3%). The extractions were performed in triplicate, as previously reported [11].

### 3.4. Microwave-Assisted Extraction (MAE)

The ground dried samples of *S. caulescens* (1.0 g) were placed into 10 mL of ethyl lactate. Then, this mixture was placed within a microwave device (Anton Parr, Aargau, Switzerland) with adjustable operating parameters (100 °C for 30 min at 10 W). Subsequently, the supernatant was centrifuged for 30 min at 9000× *g*. Finally, the solvent was filtered and evaporated under vacuum, in order to yield a dark gummy extract (50.5 ± 3.1 mg; 5.0 ± 0.3%). The extractions were performed in triplicate, as previously reported [11].

### 3.5. UHPLC-PDA-MS/MS Instrument

The Thermo Scientific Dionex Ultimate 3000 UHPLC system (Thermo Fisher Scientific, Bremen, Germany) hyphenated with a Thermo Q Exactive™ (Thermo Fisher Scientific, Bremen, Germany). Focus machine was used, as has been already reported [11]. For the analysis, 2 mg of each extract were first dissolved in 2 mL of ethanol, then filtered (PTFE filter), and finally 10 µL of the dilution was injected in the instrument, with all specifications set as previously reported [11,17].

#### LC Parameters and MS Parameters

Liquid chromatography was performed using a UHPLC C18 column (Accucore, 150 mm × 4.6 mm ID, 2.5 µm, Thermo Fisher Scientific, Bremen, Germany) operated at 25 °C. The detection wavelengths were 254, 280, 330 and 354 nm, and PDA was recorded from 200 to 800 nm for peak characterization. The mobile phases used were 1% formic acid aqueous solution (A) and 1% formic acid in acetonitrile (B). The gradient program (time (min), % (B) was set as follows: (0.00, 12); (5.00, 12); (10.00, 20); (15.00, 40); (20.00, 40); (25.00, 70); (35.00, 12). This was then coupled to 15 min for column equilibration before each injection. The flow rate was 1.00 mL min^−1^, and the injection volume was 10 µL. The standards, and the extracts dissolved in ethanol, were kept at 10 °C during storage in the autosampler. The HESI II and Orbitrap spectrometer parameters were optimized as previously reported [11,17].

### 3.6. Chemoinformatics Details

A comprehensive chemotaxonomic database was constructed encompassing all the reported secondary metabolites from twelve *Sticta* species: *S. andina, S. hypoglabra, S. cordillerana, S. gyalocarpa, S. leucoblepharis, S. parahumboldtii, S. impressula, S. ocaniesnsis, S. pseudosylvatica, S. luteocyphelata, S. lineariloba,* and *S. caulescens*. Chemical structures for 109 distinct compounds were retrieved and validated using Python v3.9.0-based chemoinformatic tools, specifically leveraging the CIRpy (https://github.com/mcs07/CIRpy, accessed on 19 January 2026) and PubChemPy (https://github.com/mcs07/PubChemPy, accessed on 19 January 2026) libraries for automated structure resolution and SMILES (Simplified Molecular Input Line Entry System) notation generation. Compound identities were cross-referenced against specialized natural product databases, including PhytoBank (https://phytobank.ca/, accessed on 5 November 2025) and BOC Sciences Bio-fermentation Database (https://bio-fermen.bocsci.com/, accessed on 5 November 2025), to ensure structural accuracy and stereochemical consistency. SMILES strings were systematically validated through canonical transformation and molecular graph reconstruction to eliminate tautomeric ambiguities and formatting inconsistencies.

Subsequent chemotaxonomic analyses employed custom Python scripts integrating multiple computational libraries. Binary presence/absence matrices were subjected to Jaccard similarity clustering, using average linkage hierarchical methods, to enable a quantitative assessment of the metabolic overlap across species [40]. This approach is particularly robust for binary ecological data, minimizing any biases that are introduced by differential metabolite abundance. Chemotaxonomic relationships and molecular scaffold diversity were interrogated using the RDKit cheminformatics toolkit, incorporating modules for fingerprint generation (rdkit.Chem.rdFingerprintGenerator), structural similarity calculations (rdkit.DataStructs), and Murcko scaffold decomposition (rdkit.Chem.Scaffolds.MurckoScaffold) [37]. Morgan fingerprints (extended-connectivity fingerprints, ECFP) were computed with radius 2 and 1024-bit vectors to capture local molecular environments and substructural features. Hierarchical clustering dendrograms were constructed via SciPy’s linkage and dendrogram functions, employing the Tanimoto distance metrics derived from the fingerprint comparisons [41]. Distance matrices were generated through squareform transformations of pairwise dissimilarity measurements, enabling the visualization of phylochemical relationships and the identification of conserved biosynthetic frameworks across the *Sticta* genus.

To assess the robustness of the chemotaxonomic clustering, a non-parametric bootstrap analysis was performed with 1000 replicates by resampling the condensed pairwise Tanimoto distance matrix with replacement [42]. Bootstrap support values (0–100%) were calculated for each internal node of the UPGMA dendrogram and are displayed directly above the branches (values ≥70% were considered to indicate a strong topological support). The cophenetic correlation coefficient (r = 0.799) was computed to evaluate how faithfully the dendrogram represents the original distance matrix.

### 3.7. Statistical Analysis

The results were expressed as the mean ± standard deviation. All statistical analyses were performed using the software GraphPad Prism 6 for Windows.

## 4. Conclusions

Conventional solvents that have been employed for the isolation of secondary metabolites over the years have evidenced that they could be toxic for both human beings and the environment. The use of ethyl lactate combined with MAE could reduce the negative environmental impact of the use of chlorinated solvents. In this sense, MAE combined with green solvents was used to produce extracts and was also compared with a conventional method, maceration, for the isolation and purification of the main compounds contained in the Chilean *S. caulescens*. The results of the chemoinformatic study allowed us to conclude that species of the genus *Sticta* exhibit great metabolic diversity and diverse and specific chemical profiles in each species. This allowed us to identify a strong chemotaxonomic differentiation across the genus, which may reflect an underlying evolutionary specialization. However, it is here strictly interpreted from the perspective of secondary metabolite diversity, confirming that secondary metabolites are powerful markers for the clear understanding of taxonomic structures, evolutionary patterns, and biosynthetic capabilities in the genus *Sticta*. Despite this heterogeneity, the preserved aromatic skeletons, depsides, and depsidones revealed a common biosynthetic basis. The species *S. caulescens* and *S. cordillerana* stand out as the most chemically complex and closely related species, sharing a greater relationship of similar metabolites and structural diversity. Their similarity places them at the center of an important chemotaxonomic lineage within the *Sticta* genus. Overall, the integration of a metabolite distribution, fingerprint similarity, and scaffold analysis confirms that secondary metabolites are informative markers for the chemotaxonomic characterization and exploration of biosynthetic diversity in the genus *Sticta*. While these results are consistent with known evolutionary patterns in the group, conclusions regarding phylogenetic relationships should be considered as hypothesis-generating and complemented with sequence-based phylogenetic analyses in future studies.

Finally, unconventional techniques together with green solvents are a promising approach in natural product chemistry that enhance safety, efficiency, and sustainability. This combination is pivotal for developing more environmentally friendly practices in academia and industry.

## Figures and Tables

**Table 1:** Secondary metabolites of the lichen *S. caulescens* extracted by maceration and microwave-assisted extraction.

Peak	t_R_ (min)	Tentative Identification	Theoretical Mass (*m*/*z*)	Measured Mass (*m*/*z*)	MS Ions (*m*/*z*)	Extraction Method	Accuracy (ppm)
Carbohydrates							
1	3.41	Manitol	181.0712	181.0710	163.0662	M, MAE	−1.1
2	3.54	Disaccharide	341.1089	341.1101	179.0560	M, MAE	3.5
Acids							
3	4.33	Gluconic acid	195.0509	195.0516	165.0454 111.0081	M, MAE	3.6
4	5.64	Citric acid *	191.0197	191.0201		M, MAE	2.1
Depsides							
11	16.76	Nor 8′-methylconstictic acid	447.0927	447.0921	209.0510 181.0516 ---	M, MAE	−1.3
17	19.96	4-O-demethylbarbatic acid	345.0974	345.0985		M, MAE	3.2
23	22.37	Fumarprotocetraric acid derivative	311.0567	311.0561		M, MAE	−1.9
Aromatics							
5	7.22	2-Ethylisophthalic acid	193.0505	193.0511	161.0291; 133.0312 123.0484 --- --- 135.0497 133.0291 137.0288 161.0283 --- 163.0442	M, MAE	3.1
6	12.91	Orsellinic acid	167.0347	167.0357		M, MAE	5.9
7	13.54	Trihydroxy benzaldehyde	153.0188	153.0199		M, MAE	7.2
8	14.84	2,4-dihydroxy benzaldehyde	137.0238	137.0231		M, MAE	−5.1
9	15.11	5,7-Dihydroxy-6-methylphthalide	179.0344	179.0353		M, MAE	5.0
10	15.26	Haematommic acid lactone	177.0191	177.0199		M, MAE	4.5
12	17.07	2-Hydroxyisophthalic acid	181.0137	181.0148		M, MAE	6.1
20	21.30	2-Ethylisophthalic acid I	193.0505	193.0515		M, MAE	5.1
24	22.56	2,4-dihydroxy benzaldehyde I	137.0238	137.0229		M, MAE	−6.5
32	26.92	Methyl orsellinate	181.0502	181.0516		M, MAE	7.7
Anthraquinones							
13	18.55	Parietin	283.0606	283.0613	135.0461 --- 149.0248	M, MAE	2.4
22	22.10	Pentahydroxymethylanthraquinone	301.0348	301.0359		M, MAE	3.6
29	25.31	Parietin I	283.0606	283.0620		M, MAE	4.9
Lipids							
14	18.71	Tetrahydroxyoctadecadienoic acid	343.2126	343.2134	--- --- --- --- --- --- --- --- --- ---	M, MAE	2.3
15	19.28	4,5-Dihydroxy-2-nonenoic acid	187.0974	187.0987		M, MAE	6.9
21	21.95	Dihydroxydecenoic acid	201.1132	201.1139		M, MAE	3.4
25	22.82	Trihydroxyoctadecadienoic acid	327.2177	327.2181			2.2
26	23.31	Trihydroxyoctadecenoic acid	329.2328	329.2320		M, MAE	−2.4
28	24.11	Pentahydroxytetracosanoic acid	447.3322	447.3337		M, MAE	3.3
34	27.59	Dihydroxyoctadecenoic acid	313.2390	313.2399		M, MAE	2.8
35	29.51	9-hydroxyoctadecatrienoic acid	293.2117	293.2121		M, MAE	1.4
36	29.71	Pentahydroxytetracosanoic acid I	447.3322	447.3329		M, MAE	1.6
37	30.58	Hydroxyoctadecadienoic acid	295.2273	295.2284		M, MAE	3.7
Dibenzofurans							
16	19.30	Usnic acid *	343.0818	343.0825	---	M, MAE	2.0
Depsidones							
19	20.84	Hypostictic acid isomer	371.0778	371.0789	327.0987; 179.0391 285.0855	M, MAE	2.9
18	20.56	Didechlorolecideoidin	329.0661	329.0672		M, MAE	3.3
Pulvinic acid and derivatives							
33	27.51	Pulvinic acid derivative	323.0556	323.0550	117.0340	MAE	−1.8
Triterpenes							
31	26.84	Retigeric acid derivative	487.3429	487.3440	---	M, MAE	2.2
30	26.50	Retigeric acid B	501.3216	501.3228	---	M, MAE	2.4
27	23.69	Retigeric acid derivative	555.2963	555.2969	---	M, MAE	1.1

Abbreviations: T_R_ = Retention time, M = Maceration, MAE = Microwave-Assisted Extraction. * Identified by spiking and recovery experiments with an authentic compound.

## Data Availability

The original data of this study are contained within the article and Supplementary Materials.

## Supplementary Materials

The following supporting information can be downloaded at: https://www.mdpi.com/article/10.3390/plants15111761/s1, Figure S1: The absence and presence of molecules in the 12 different species of *Sticta* lichens is shown. These analyses are derived from the digital database, where their SMILES and the species to which they belong can be found; Figure S2–S12: Predominant ring-containing scaffolds identified in the *S. andina*, *S. cordillerana*, *S. gyalocarpa*, *S. hypoglabra*, *S. impressula*, *S. leucoblepharis*, *S. lineariloba*, *S. luteocyphelata*, *S. ocaniensis*, *S. parahumboldtii*, *S. speudosylvatica*, respectively. Table S1: Identification of metabolites in 12 species of the genus Sticta from Colombia and Chile.
