# Supplementary material for: Phytochemical Profiling of Sticta caulescens De Not.: Green Extraction and Multiscale Chemotaxonomic Analysis
Source: Plants (Basel). 2026 Jun 5;15(11):1761. doi: 10.3390/plants15111761 (PMC13259522; doi:10.3390/plants15111761)
Supplement: Supplementary file 1 [file plants-15-01761-s001.zip › plants-4320231-supplementary.pdf]

Phytochemical Profiling of *Sticta caulescens* De Not.: Green Extraction and Multiscale Chemotaxonomic Analysis

Nicolás Cifuentes-Araya [1], Diego Valdivia [1], Mariano Walter Pertino [2], Daniela Marroquín-Guerra [3], Osvaldo Yáñez [4], Olimpo García-Beltrán [5,6], Alejandro Ardiles [7,*] and Carlos Areche [1,*]

[1] Departamento de Química, Facultad de Ciencias, Universidad de Chile, Casilla 653, Santiago 8320000, Chile; nicocifuentesaraya@gmail.com (N.C.-A.); diego.valdivia@ug.uchile.cl (D.V.)

[2] Laboratorio de Química de Productos Naturales, Instituto de Química de Recursos Naturales, Universidad de Talca, Casilla 747, Talca 3460000, Chile; mwalter@utalca.cl

[3] Facultad de Ciencias, Ingeniería e Innovación, Universidad de Ibagué, Carrera 22 Calle 67, Ibagué 730002, Colombia; 4220211021@estudiantesunibague.edu.co

[4] Centro de Modelación Ambiental y Dinámica de Sistemas (CEMADIS), Facultad de Ingeniería y Negocios, Universidad de Las Américas, Santiago 7500975, Chile; oyanez@udla.cl

[5] Centro de Estudios e Investigación en Salud y Sociedad (EISS), Universidad Bernardo O'Higgins, General Gana 1702, Santiago 8370854, Chile; jose.garcia@unibague.edu.co

[6] Co-Laboratorio de Investigación en Bioeconomía Regional, Universidad de Ibagué, Carrera 22 Calle 67, Ibagué 730002, Colombia

[7] Departamento de Ciencias Básicas, Facultad de Ciencias, Universidad Santo Tomas, Antofagasta 1240000, Chile

* Correspondence: aardiles2@santotomas.cl (A.A.); areche@uchile.cl (C.A.)

Table of contents

Contents	Page
**Table S1**. Identification of metabolites in 12 species of the genus *Sticta* from Colombia and Chile	3
**Figure S1**. The absence and presence of molecules in the 12 different species of Sticta lichens is shown. These analyses are derived from the digital database, where their SMILES and the species to which they belong can be found.	11
**Figure S2**. Predominant ring-containing scaffolds identified in the *S. andina*. The frequency of each scaffold (as a percentage).	12
**Figure S3**. Predominant ring-containing scaffolds identified in the S. cordillerana. The frequency of each scaffold (as a percentage).	12
**Figure S4**. Predominant ring-containing scaffolds identified in the *S. gyalocarpa*. The frequency of each scaffold (as a percentage).	13
**Figure S5**. Predominant ring-containing scaffolds identified in the *S. hypoglabra*. The frequency of each scaffold (as a percentage).	13
**Figure S6.** Predominant ring-containing scaffolds identified in the *S. impressula*. The frequency of each scaffold (as a percentage).	14
**Figure S7.** Predominant ring-containing scaffolds identified in the *S. leucoblepharis*. The frequency of each scaffold (as a percentage).	14
**Figure S8**. Predominant ring-containing scaffolds identified in the *S. leariloba*. The frequency of each scaffold (as a percentage).	15
**Figure S9.** Predominant ring-containing scaffolds identified in the *S. luteocyphelata*. The frequency of each scaffold (as a percentage).	15
**Figure S10.** Predominant ring-containing scaffolds identified in the *S. ocaniesnsis*. The frequency of each scaffold (as a percentage).	16
**Figure S11.** Predominant ring-containing scaffolds identified in the *S. parahumboldtii*. The frequency of each scaffold (as a percentage).	16
**Figure S12**. Predominant ring-containing scaffolds identified in the *S. speudosylvatica*. The frequency of each scaffold (as a percentage).	17

Table S1. Identification of metabolites in 11 species of the genus *Sticta* from Colombia and Chile

Peak	Tentative identification	[M-H]-	Retention time (min)	Theorical mass (*m/z*)	Measured mass (*m/z*)	Accuracy (ppm)	Metabolite type	MS ions (ppm)	Lichen
1	Gluconic Acid	$C_6H_{11}O_7$	1.28	195.0509	195.0505	2.0	Acid	165.0401;	2; 3; 7; 9; 10; 11; 12
2	Unknown	$C_{11}H_5O_5N$	1.28	231.0184	231.0195	-4.8	-	---	6
3	Manitol	$C_6H_{13}O_6$	1.31	181.0712	181.0714	-1.1	Carbohidrate	181.0717; 163.0606	7; 9; 10; 11; 12
4	Arabic acid	$C_5H_9O_6$	1.29	165.0399	165.0401	-1.2	Acid	147.0293; 113.0237; 129.0196	6
5	Citric Acid	$C_6H_7O_7$	1.38	191.0196	191.0192	2.0	Acid	111.0080	1; 2; 3; 4; 5; 6; 7; 8; 9; 11; 12
6	Unknown	$C_{15}H_5O_3N_2$	1.38	261.0289	261.0300	-4.2	--	---	1; 7; 8; 10
7	4-ethyl-2-Ethylisophthalic acid	$C_{10}H_9O_4$	1.44	193.0504	193.0501	1.6	A	133.0288	1; 2; 3; 4; 6
8	Unknown	$C_8H_{10}O_5N$	1.56	200.0563	200.0559	2.0	-	--	1; 3; 4; 6
9	Unknown	$C_{15}H_5O_3N_2$	1,64	261.0247	261.0278	-11.8	-	---	7; 8; 10
10	Isocitric Acid	$C_6H_7O_7$	1.77	191.0195	191.0192	1.6	Acid	111.0079;	1; 2; 3; 4; 6; 7
11	Unknown	$C_7H_9O_7$	1.91	205.0352	205.0348	2.0	-	187.0245; 173.0087; 121.1131	2
12	Unknown	$C_7H_7O_6$	2.24	187.0246	187.0243	1.6	-	125.0237; 137.2503;	2
13	2-Ethylisophthalic acid	$C_{10}H_9O_4$	2.85	193.0505	193.0501	2.0	A	161.0240; 133.0290	1; 2; 3; 4; 6; 7; 8; 9; 10; 12
	Trehalose	$C_{12}H_{22}O_{11}$	3.54	341.1089	341.1101		Carbohidrate	179.0560	12
14	Trihydroxy benzaledehyde	$C_7H_5O_4$	4.81	153.0188	153.0189	-0.7	A	137.0238	1; 2; 3; 4
15	2-Hydroxyisophthalic acid	$C_8H_5O_5$	7.63	181.0137	181.0141	-2.2	A	137.0238	4; 12
16	2,4-dihydroxy benzaldehyde	$C_7H_5O_3$	8.02	137.0238	137.0239	-0.7	A	121.0289	1; 2; 3; 4
17	4-O-Demethylglomellic acid	$C_{24}H_{25}O_9$	8.65	457.1476	457.1499	-5.0	d	---	5; 8
18	Unknow	$C_{22}H_{23}O_7$	8.77	399.1444	399.1413	7.8	-	---	8
19	Unknow	$C_7H_{11}O_5$	8.86	175.0606	175.0611	-2.9	-	---	5
20	Grayanic acid	$C_{23}H_{25}O_7$	8.91	413.1600	413.1569	7.5	d	181.06503	8
21	Unknown	$C_{13}H_{16}O_7N$	8.98	298.0940	298.0927	4.4	-	175.0609; 283.0210; 202.0696;	5
22	Unknown	$C_{11}H_9O_7$	9.33	253.0361	253.0348	5.1	-	137.0603; 181,0505; 249.3808; 173.4203;	5
23	Atranol	$C_8H_7O_3$	9.44	151.0395	151.0396	-0.7	A	123.0445; 135.0445	3
24	Unknown	$C_{24}H_{23}$ O_8	9.48	439.1371	439.1393	-5.0	-	151.0397; 105.0948; 121.4871;	5

25	5,7-Dihydroxy-6-methylphthalide	$C_9H_7O_4$	9.64	179.0344	179.0347	-1.6	A	135.0444; 107.0494	4; 5; 7; 8; 10; 12
26	Unknown	$C_{16}H_{15}O_{10}$	9.73	367.0665	367.0639	7.1	-	---	1; 4
27	Unknown	$C_{30}H_{27}O_{13}N$	10.03	609.1475	609.1482	-1.1	-	---	1; 4; 6
28	Unknow	C18H15O4	10.31	295.0970	295.0935	11.8	-	---	9
29	Unknow	$C_{17}H_{13}O_6$	11.31	313.0712	313.0724	-3.8	--	---	1; 2; 3; 4; 5
30	1,5-Pentanedicarboxylic acid	$C_7H_{11}O_4$	10.56	159.0657	159.0660	-1.9	L	115.0758	5
31	Unknown	$C_{29}H_{25}O_{13}N$	10.54	595.1316	595.1326	-1.7	-	---	3
32	Didechlorolecideoidin	$C_{17}H_{13}O_7$	10.64	329.0661	329.0676	-4.6	D	209.0456; 285.0776; 151.0396; 179.0347; 123.0443	3, 12
33	Decahydroxyoxopentacosanoic acid	$C_{25}H_{47}O_{13}$	10.71	555.3017	555.3047	-5.4	L	---	8
34	Orsellinic acid	$C_8H_7O_4$	11.04	167.0347	167.0344	1.8	A	123.0442	2; 3; 4; 5; 6; 12
35	Unknow	$C_{21}H_{19}O_{12}$	11.04	463.0877	463.0893	-3.4	-	---	3
36	Unknow	$C_{10}H_9O_5$	11.07	209.0450	209.0458	-3.8	-	---	5; 6
37	Nor 8´-methylconstictic acid	$C_{21}H_{19}O_{11}$	11.11	447.0927	447.0942	-3.4	d	209.0455	2
38	Unknow	$C_{17}H_{13}O_6$	11.20	313.0712	313.0720	-2.5	-	---	7
39	Metil-2,6-dihidroxibenzoate	$C_8H_7O_4$	11.21	167.0344	167.0346	-1.1	A	109.0287; 137.0238	9
40	Hypostictic acid isomer	$C_{19}H_{15}O_8$	11.36	371.0778	371.0782	-1.1	D	195.0665; 327.0885; 341.0679; 179.0347	1; 3; 4; 7; 12
41	Unknow	$C_{19}H_{16}O_9N$	11.69	402.0825	402.0841	-3.98	-	---	11
42	Fumarprotocetraric acid derivative	$C_{17}H_{11}O_6$	11,70	311.0556	311.0564	-2.5	d	---	7; 10: 12
43	4,5-Dihydroxy-2-nonenoic acid	$C_9H_{15}O_4$	12.11	187.0974	187.0977	-1.6	L	171.1025; 143.1072	1; 2; 3; 4; 9; 10; 12
44	2,4-Dicarboxy-3-hydroxy-5-methoxytoluene	$C_{10}H_9O_6$	12.14	225.0407	225.0399	3.5	A	181.0503; 167.0346; 149.0240	4
45	Unknown	$C_{17}H_{13}O_6$	12.16	313.0724	313.0727	-1.0	-	---	4
46	Unknown	$C_{18}H_{15}O_7$	12.37	343.0818	343.0826	-2.3	-	---	7; 9
47	Unknown	$C_{21}H_{17}O_{12}$	12.40	461.0737	461.0720	3,7	-	---	6
48	Unknown	$C_{20}H_{17}O_8$	12.52	385.0939	385.0923	4,1	-	---	1; 2; 3
49	Unknow	$C_{10}H_9O_4$	12.81	193.0501	193.0502	-0,5	-	---	1; 2; 3; 4; 5; 6 ; 7; 8; 9; 10
50	Unknow	$C_{27}H_{45}O_6$	13.04	465.3216	465.3231	-3,2	-	---	3
51	2,4-dihydroxy benzaldehyde	$C_7H_5O_3$	13.07	137.0237	137.0239	-1,5	A	121.0288	5; 12
52	Consalizinic acid derivative I	$C_{19}H_{13}O_{11}$	13.36	417.0458	417.0474	-3,8	D	373.0573; 387.0373; 225.0406, 177.0193	11
53	4-Ethoxy-3-formyl-2-hydroxy-6-methylbenzoic acid	$C_{11}H_{11}O_5$	13.41	223.0614	223.0606	3,6	A	177.0190; 133.0296;	1; 2; 3; 4; 7; 9; 10
54	Unknow	$C_{20}H_{15}O_8$	13.47	383.0767	383.0781	-3,6	-	---	1
55	Consalizinic acid derivative II	$C_{20}H_{17}O_{11}$	13,47	433.0771	433.0787	-3,7	D	401.0524; 417.0474; 373.0574	11
	Trihydroxy benzaldehyde	$C_7H_6O_4$	13.54	153.0188	153.0199			---	12
56	Cynodontin or Citreorosein isomer,	$C_{15}H_9O_6$	13.71	285.0399	285.0410	-3.9	Anthraquinone	151.0396; 137.0237	2

57	Consalizinic acid derivative I isomer	$C_{19}H_{13}O_{11}$	13.78	417.0458	417.0474	-3.84	D	373.0573; 343.0467, 77.0190; 401.0523	10
58	Unknow	$C_{30}H_{47}O_7$	13.79	519.3322	519.3337	-2.9	-	---	3
59	1,4,5,6,8-Pentahydroxy-3-ethylanthraquinone	$C_{15}H_9O_7$	13.87	301.0348	301.0361	-4.3	Anthraquinone	---	4
60	Unknow	$C_{19}H_{15}O_4$	13.87	307.0970	307.0939	10.0	-	---	8
61	Unknow	$C_{14}H_{13}O_7$	13.99	293.0661	293.0674	-4.4	-	---	3
62	Haemathamnolic acid isomer	$C_{19}H_{15}O_{10}$	14.18	403.0665	403.0681	-3.97	D	359.0781; 371.0414; 209.0455	11
63	Fumarprotocetraric acid derivative	$C_{17}H_{11}O_6$	14.78	311.0567	311.0569	-0.6	d	---	1; 2; 3; 7; 10
64	Constictic acid	$C_{19}H_{13}O_{10}$	14.61	401.0509	401.0528	-4.74	D	357.0625; 313.0726 ; 283.0619 ; 255.0670; 121.0289	11
65	Hypostictic acid isomer	$C_{19}H_{15}O_8$	14.98	371.0781	371.0767	3,8	D	327.0883; 195.0664; 179.0347	5
66	Thelephoric acid	$C_{18}H_7O_8$	14.81	351.0141	351.0154	-3.7	Terphenylquinones	---	3
67	Methylstictic acid	$C_{20}H_{15}O_9$	15.26	399.0716	399.0728	-3.0	D	371.0779; 193.0504	2; 7
	Haematommic acid lactone	$C_9H_8O_5$	15.27	177.0191	177.0199			133.0291	12
68	Nor 8´-metilconstictic acid	$C_{21}H_{19}O_{11}$	15.28	447.0927	447.0944	-3.80	D	401.0524; 209.0455	11, 12
69	Protocetraric acid	$C_{18}H_{13}O_9$	17.17	373.0560	373.0574	-3.75	D	355.0468; 329.0674; 311.0568; 227.0352; 267.0669; 285.0777	11
70	Hypoconstictic acid	$C_{19}H_{15}O_9$	17.27	387.0716	387.0729	-3.4	D	267.0673; 311.0552; 149.0238; 343.0827;167.0345	3
71	Unknow	$C_{14}H_{13}O_6$	17.84	277.0712	277.0724	-4.3	-	--	3; 6
72	12,13,15-Trihydroxy-9-octadecenoic acid	$C_{18}H_{15}O_5$	18.11	329.2328	329.2340	-3.6	L	285.1716;	2; 6
73	Unknow	$C_{20}H_{13}O_8$	18.19	381.0610	381.0626	-4.2	-	---	3; 4
74	Salazinic acid	$C_{18}H_{11}O_{10}$	18.21	387.0352	387.0368	-4.13	D	343.0468; 269.0458; 241.0507; 325.0365; 299.0569	11
75	Unknown	$C_{10}H_9O_4$	18.45	193.0505	193.0501	2.1	-	---	3; 4
76	Unknown	$C_{30}H_{47}O_7$	18.61	519.3322	519.3319	0.6	-	--	3
	Tetrahydroxyoctadecadienoic acid	$C_{18}H_{36}O_6$	18.71	343.2126	343.2134			---	12
77	Menegazziaic acid	$C_{18}H_{13}O_9$	18.79	373.0560	373.0575	-4.0	D	311,0570; 255.0666; 329.0679	3
78	Norstictic acid	$C_{18}H_{11}O_9$	18.86	371.0403	371.0417	-3.8	D	327.0526; 151.0396; 123.0444;	3
79	Unknow	$C_{22}H_{19}O_{10}$	18.88	443.0978	443.0996	-4.1	-	---	3
80	Physodalic acid	$C_{20}H_{15}O_{10}$	18.99	415.0665	415.0681	-3.85	D	359.0417; 315.0520; 343.0832; 387,0367; 373.0573; 401.0525	11
81	Unknow	$C_{26}H_{19}O_{10}$	19.00	491.0978	491.0997	-3.9	-	---	3
82	Derivative Methyl 8-hydroxy-4-0-demethylbarbatate	$C_{19}H_{19}O_9$	19.05	391.1045	391.1029	4.1	d	359.0788	3
83	12,13,15-Trihydroxy-9-octadecenoic acid	$C_{18}H_{33}O_5$	19.05	329.2328	329.2336	-2.4299	L	---	7

84	Haemoventosin	$C_{15}H_{11}O_7$	19.16	303,0519	303.0505	4.7	Naphthaquinone	259.0619; 231.0667; 189.0560;	3
85	α-acetilconstictic acid derivative I	$C_{21}H_{17}O_{11}$	19.22	445.0771	445.0786	-3.3	D	415.0680; 371.0780; 427.0676; 343.0830;193.0504, 401.0522	11
86	Conhypoprotocetraric acid or Convirensic acid	$C_{18}H_{15}O_8$	19.25	359.0781	359.0767	3.9	D	344.0545; 302.0442	3
87	4-O-dimethylbaemycesic acid	$C_{18}H_{15}O_8$	19.27	359.0781	359.0767	3.9	d	181.0714; 163.0397; 137.0236	1; 2; 3;5; 6
88	Orsellinic acid Isomer	$C_8H_7O_4$	19.45	167.0344	167.0346	-1.1974	A	123.0440; 149.0235	9; 10
89	Lecanoric acid	$C_{16}H_{13}O_7$	19.51	317.0661	317.0671	0.6	d	167.0345; 123.0443; 149.0238;	1; 2; 3; 4; 5; 6
90	Constictic acid isomer	$C_{19}H_{13}O_{10}$	19.56	401.0509	401.0524	-3.74	D	357,0626; 313.0726; 343.0831; 255.0622	11
91	Pentahydroxytetracosanoic acid	$C_{24}H_{47}O_7$	19.67	447.3322	447.3336	-3.1	L	---	1; 3; 7; 9; 10; 12
92	2-Methyl-5-hydroxy-6-hydroxymethyl-7 -Methoxychromone	$C_{12}H_{11}O_5$	19.73	235.0606	235.0615	-3.8	C	181.0504	3
93	Unknown	$C_{20}H_{17}O_8$	19.79	385.0939	385.0923	4.1	-	---	1; 4; 5; 10
94	Heptahydroxytrioxooctadecanoic acid	$C_{18}H_{29}O_{12}$	19.86	437.1664	437.1645	4.3	L	---	1; 4; 5; 6; 7; 9; 10
95	5,7-Dihydroxy-6-methylphthalide derivative	$C_9H_7O_3$	19.88	163.0395	163.0392	1.8	A	119.0492	10
	4-O-demethylbarbatic acid	$C_{18}H_{18}O_7$	19.96	345.0974	345.0985			181.0516	12
96	Criptostictic acid derivative	$C_{18}H_{11}O_8$	20.04	355.0454	355.0462	-2.2	D	133.0288; 239.0715; 311.0572; 179.0345;	7
97	Unknow	$C_{18}H_{17}O_6$	20.08	329.1025	329.1032	-2.1	-	---	9
98	Unknow	$C_{20}H_{15}O_8$	20.16	383.0767	383.0775	-2.0	-	---	9; 10
99	Unknow	$C_{21}H_{19}O_9$	20.18	415.1045	415.1029	3.9	-	---	3; 5; 9; 10
100	Unknow	$C_{28}H_{23}O_{11}$	20.12	535.1240	535.1257	-3.1	-	---	3
101	Unknow	$C_{15}H_{13}O_3$	20.13	241.0872	241.0874	-0.8	-	----	1; 2
102	Heptahydroxytetraoxoicosanoic acid	$C_{20}H_{31}O_{13}$	21.19	479.1765	479.1746	3.9	L	---	7
103	Tetrahydroxytricosanoic acid	$C_{23}H_{45}O_6$	20.26	417.3232	417.3216	3.9	L	403.3073	1; 3; 4; 7
104	Tetrahydroxytrioxoundecanoic acid	$C_{11}H_{15}O_9$	20.30	291.0716	291.0699	5.8	L	---	8
105	Stictic acid	$C_{19}H_{13}O_9$	20.34	385.0560	385.0576	-4.1	D	341.0674; 357.0622; 297.0774; 313.0721; 193.0504; 269.0826	11
106	Parietin	$C_{16}H_{11}O_5$	20.39	283.0606	283.0617	-3.9	Antraquinone	179.0345	1; 2; 6; 12
107	Unknow	$C_{24}H_{47}O_{11}N_2$	20.39	539.3157	539.3180	-4.3	-	---	3
108	Evernic acid isomer	$C_{17}H_{15}O_7$	20.46	331.0818	331.0830	-3.6	d	167.0347; 123.0447; 149.0240	1
109	Hypoconstictic acid	$C_{19}H_{15}O_9$	20.50	387.0716	387.0732	-4.1	D	149.0238; 343.0836; 167.0345	4
110	Cryptostictic acid	$C_{19}H_{15}O_9$	20.50	387.0716	387.0725	-2.3	D	267,0661; 343,0825; 311.05067; 239.0710	7; 8
111	Retigeric acid derivative	$C_{30}H_{43}O_7$	20.51	515.3009	515.3025	-3.1	Triterpene	---	3; 12
112	Retigeric acid B	$C_{30}H_{45}O_6$	20.56	501.3216	501.3236	-4.0	Triterpene	---	3; 12
113	Salazinic acid isomer	$C_{18}H_{11}O_{10}$	20.58	387.0352	387.0368	-4.13	D	343.0468; 299.0565	11

114	Unknown	$C_{23}H_{22}O_{10}N$	20.68	472.1244	472.1259	-3.2	-	-----	6
115	9,10-dihydroxyoctadecatrienoic acid	$C_{18}H_{29}O_4$	20.69	309.2081	309.2066	4.9	L	291.1975	1; 2; 4
116	Unknow	$C_{17}H_{13}O_6$	20.84	313.0712	313.0727	-4.8	-	----	1; 2; 6
117	Pulvinic acid derivative I	$C_{18}H_{11}O_6$	20.97	323.0556	323.0556	0.0	Pulvinic acid y derivates	133.0286; 117.0335	10; 12
118	9,10,12 trihydroxytriacontaheptaenoic acid	$C_{30}H_{45}O_5$	20.99	485.3284	485.3267	3.5	L	---	3
119	4-0-Demethylbarbatic acid	$C_{18}H_{17}O_7$	20.99	345.0974	345.0989	-4.3	d	181.0505; 163.0396; 137.0603	4
120	Unknow	$C_{24}H_{23}O_{10}N$	21.02	485.1322	485.1319	0.6	-	---	1; 5
121	Methyl orsellinate	$C_9H_9O_4$	21.05	181.0502	181.0501	0.5	A	163.0389	1; 12
122	Heptahydroxyetraoxoicosanoic acid	$C_{20}H_{31}O_{13}$	21.17	479.1752	479.1765	-2.7	L	---	1; 2; 3; 4; 5; 6; 9
123	Gyrophoric Acid	$C_{24}H_{19}O_{10}$	21.25	467.0991	467.0978	2.8	d	167.0346; 317.0673; 123.0445; 149.0238;	1; 3; 4; 6; 11
124	Galbinic acid	$C_{20}H_{13}O_{11}$	21.27	429.0458	429.0474	-3.73	D	403.0681; 371.0417; 401.0524; 327.0518; 149.0239	11
125	Hyposalazinic acid	$C_{18}H_{13}O_8$	21.28	357.0610	357.0623	-3.6	D	313.0723; 135.0444; 179.0348	1
	2-Ethylisophthalic acid I	$C_{10}H_{10}O_4$	21.30	193.0505	193.0515			161.0283	12
126	Hydroxytetracosapentaenoic acid	$C_{24}H_{37}O_3$	21.42	373.2743	373.2743	0.0	L	---	10
127	Orsellinic acid isomer	$C_8H_7O_4$	21.47	167.0347	167.0344	1.8	A	149.0239; 123.0443	1; 4; 6
128	Dihydroxyoctadecenoic acid	$C_{18}H_{33}O_4$	21.48	313.2390	313.2395	-1.6	L	----	2; 5; 6; 12
129	Norstictic acid	$C_{18}H_{11}O_9$	21.64	371.0403	371.0417	-3.77	D	27.0517; 227.0716; 151.0390; 243.0297	11
130	Dihydroxyoctadec-6-enoic acid	$C_{18}H_{33}O_4$	21.58	313.2379	313.2379	0.0	L	---	10
131	Loxodinol isomer	$C_{25}H_{29}O_9$	21.64	473.1812	473.1818	-1.2	DE	429.1919	9
132	EthyI 2,4-dihydroxy-6-n-nonylbenzoate	$C_{18}H_{27}O_4$	21.65	307.1909	307.1922	-4.2	A	263.1659	1; 2; 3; 4
133	Evernic Acid	$C_{17}H_{15}O_7$	21.81	331.0828	331.0818	3.0	d	167.0345; 123.0444; 149,0238;	1; 2; 3; 4; 5
134	Protocetraric acid Isomer	$C_{18}H_{13}O_9$	21.85	373.0560	373.0573	-3.48	D	355.0460; 329.0674; 285.0780, 311.0567; 255.,0672	11
135	Unknown	$C_{22}H_{22}O_8N$	21.83	428.1360	428.1345	3.5	-	----	6
136	Strepsilin	$C_{15}H_9O_5$	21.89	269.0450	269.0462	-4.5	DBF	225.0554	2; 5
	Dihydroxydecanoic acid		21.95	201.1132	201.1139				12
137	Unknown	$C_{30}H_{29}O_4$	21.97	453.2066	453.2061	1.1	-	---	3
138	Unknown	$C_{18}H_{11}O_6$	22.01	323.0556	323.0570	-4.33	-	---	11
	Pentahydroxymethylanthraquinone	C10H20O4	22.10	301.0348	301.0359				12
139	Hexahydroxytrioxooctacosatrienoic acid	$C_{28}H_{43}O_{11}$	22.12	555.2805	555.2841	-6.4832	L	---	9

140	Nonahydroxyoctacosatetraenoic acid	$C_{28}H_{47}O_{11}$	22.26	559.3124	559.3132	-1.4	L	---	2
141	Unknow	$C_{28}H_{41}O_9N_2$	22.26	549.2849	549.2812	6.8	-	---	3
142	Norsolorinic acid	$C_{20}H_{17}O_7$	22.44	369.0974	369.0989	-4.06	-	---	11
143	Unknow	$C_{25}H_{33}O_{13}$	22.46	541.1921	541.1909	2.2	-	---	1; 2; 3; 6; 10
144	Hydroxytetracosapentaenoic acid derivative	$C_{24}H_{37}O_3$	22.61	373.2743	373.2741	0.5	-	---	10
145	Hydroxytrioxotricosanoic acid	$C_{23}H_{39}O_6$	22.53	411.2747	411.2757	-2.4	L	---	8
	2,4-dihydroxy benzaldehyde I	$C_7H_6O_3$	22.56	137.0238	137.0229			---	12
	Trihydroxyoctadecadienoic acid	$C_{18}H_{32}O_5$	22.82	327.2177	327.2181			---	12
146	Squamatic acid	$C_{19}H_{17}O_9$	22.89	389.0873	389.0886	-3.3	d	343.0836; 163.0396; 193.0139; 149.0238; 121.0286	1; 3; 4
147	Picrolichenic acid	$C_{25}H_{29}O_7$	22.72	441.1913	441.1926	-3.0	Depsones	---	1
148	Heptahydroxydioxohexacosanoic acid	$C_{26}H_{47}O_{11}$	22.76	535.3118	535.3134	-3.0	L	---	6
149	Unknow	$C_{30}H_{45}O_4$	22.83	469.3319	469.3335	-3.4	-	---	3
150	2,2' -Di-O-methylanziaic acid	$C_{26}H_{33}O_7$	22.85	457.2226	457.2244	-3.9	d	413.2345;	4
151	Dihydroxytetracosahexaenoic acid	$C_{24}H_{35}O_4$	22.85	387.2535	387.2552	-4.4	L	---	5
152	Hydroxyoctadecadienoic acid	$C_{18}H_{31}O_3$	22.90	295.2273	295.2273	0.0	L	---	10; 12
153	Orsellinic acid Isomer	$C_8H_7O_4$	22.92	167.0344	167.0348	-2.3	A	149.0240; 123.0445	11
154	Pulvinic acid	$C_{18}H_{11}O_5$	22.98	307.0606	307.0613	-2.2	Pulvinic acid y derivates	117.0338; 263.0713	9
155	4-0-Demethylbarbatic acid	$C_{18}H_{17}O_7$	23.02	345.0974	345.0986	-3.5	d	123.0443; 137.0237; 181.0502	1
156	Psoromic acid	$C_{18}H_{13}O_8$	23.06	357.0610	3570626	-4.4	D	313.0726; 181.0502; 179.0347; 327.0520; 269.0826; 285.0776	11
157	Methylgyrophoric acid	$C_{25}H_{21}O_{10}$	23.15	481.1135	481.1147	-2.5	d	149.0238; 123.0442; 167.0346; 317.0671	1; 4
158	Evernic acid isomer	$C_{17}H_{15}O_7$	23.22	331.0818	331.0832	-4.2	d	149.0239; 123.0443; 167.0346; 105.0337	11
159	Skyrin	$C_{30}H_{17}O_{10}$	23.28	537.0822	537.0840	-3.4	Anthraquinones	----	3
	Trihydroxyoctadecenoic acid	$C_{18}H_{34}O_5$	23.31	329.2328	329.2320		---		
160	Angardianic acid	$C_{19}H_{35}O_4$	23.36	327.2543	327.2547	-1.2	Acids	283.2649; 309.2081	2; 4
161	Pentadecatetraenoic acid	$C_{15}H_{21}O_2$	23.38	233.1542	233.1545	-1.2	L	---	9; 10
162	9-hydroxyoctadecatrienoic acid	$C_{18}H_{29}O_3$	23.45	293.2117	293.2130	-4.4	L	277.2180	6; 12
163	Unknow	$C_{18}H_{15}O_7$	23.53	343.0818	343.0824	-1.7	-	---	9

164	Pulvinic acid derivative II	$C_{19}H_{13}O_5$	23.68	321.0763	321.0770	-2.1	Pulvinic acid y derivates	117.0337	9; 10; 11
165	Pulvinic acid	$C_{18}H_{11}O_5$	23.77	307.0606	307.0620	-4.5	Pulvinic acid y derivates	263.0720; 117.0339	11
166	Furfuric acid isomer	$C_{28}H_{23}O_{12}$	23.82	551.1190	551.1197	-1.2	D	371.0784; 193.0504; 179.0347; 207.0297; 193.0504	8
167	Unknow	$C_{26}H_{47}O_5N_2$	23.82	467.3485	467.3492	-3,9	-	---	3
168	Unknow	$C_{30}H_{27}O_6$	23.89	483.1808	483.1820	-2,5	-	---	1
169	Unknow	$C_{30}H_{25}O_6$	24.01	481.1651	481.1663	-2,5	-	---	1
170	Unknow	$C_{15}H_{13}O_3$	24.02	241.0872	241.0872	0,0	-	---	1
171	Trihydroxyheptacosa pentaenoic acid	$C_{27}H_{43}O_5$	24.05	447.3110	447.3127	-3.8	L	---	8
172	Barbatic Acid	$C_{19}H_{19}O_7$	24.26	359.1141	359.1131	2.8	d	137.0603; 163.0396; 181.0509	1; 4
173	Hydroxytrioxodocosanoic acid	$C_{22}H_{37}O_6$	24.29	397.2590	397.2601	-2.8	L	---	8
174	Thamnolic acid isomer	$C_{19}H_{15}O_{11}$	24.41	419.0614	419.0630	-3.8	d	375.0730; 167.0344; 209.0455; 181.0503	11
175	Orsenillic acid derivated II	$C_8H_7O_4$	24.73	167.0344	167.0347	-1.80	-	149.0239; 1230444;	11
176	Unknow	$C_{26}H_{33}O_8$	24.73	473.2190	473.2175	3.2	-	---	2
177	Lobaric acid	$C_{25}H_{27}O_8$	24.81	455.1706	455.1718	-2.6	D	411.1824; 367.1811	1
178	Unknow	$C_{22}H_{27}O_7$	24.97	403.1770	403.1757	3.2	-		2
179	Unknow	$C_{30}H_{41}O_8$	25.27	529.2819	529.2801	3.4	-	---	4
	Parietin I	$C_{16}H_{12}O_5$	25.31	283.0606	283.0620			149.0248	12
180	Hypothamnolic acid	$C_{19}H_{17}O_{10}$	25.49	405.0822	405.0832	-2.5	d	209.0456; 181.0499	1
181	Unknow	$C_{25}H_{11}O_7$	25.53	423.0505	423.0497	1.9	-	---	4
182	Pulvinic acid derivative III	$C_{19}H_{13}O_5$	25.67	321.0763	321.0777	-4.3	Pulvinic acid y derivates	117.0338	11
183	Dihydroxyicosahexaenoic acid	$C_{20}H_{27}O_4$	26.02	331.1909	331.1925	-4.8	L	-----	11
184	Usnic acid	$C_{18}H_{15}O_7$	26.05	343.0818	343.0831	-3.8	DBF	231.0658; 328.0585; 259.0604	3; 4; 6; 7; 8; 12
185	Nephromopsic acid or Roccellaric acid	$C_{19}H_{33}O_4$	26.32	325.2392	325.2379	4.0	Acids	281.2494	3; 4
186	Unknow	$C_{28}H_{25}O_5N$	26.87	455.1733	455.1723	2.20	-	---	11
187	Perlatolic acid	$C_{25}H_{31}O_7$	26.98	443.2070	443.2078	-1.8	d	205.0867; 179.1073; 223.0973	7; 8
188	Caperatic acid	$C_{21}H_{37}O_7$	28.14	401.2539	401.2549	-2.4	Acids	255.2327	8
189	Atranorin	$C_{19}H_{17}O_8$	29.64	373.0923	373.0937	-3.75	d	177.0192; 163.0397	9; 11

190	Pentahydroxytetracosanoic acid I	$C_{24}H_{53}O_7$	29.71	447.3322	447.3329	---

A = Aromatic; L = Lipid; D= depsidone; d = depside; DE = diphenylether; DBF = dibenzofuran. C = Chromone
1=*S. andina*; **2**=*S. hypoglabra*; **3**=*S. cordillerana*; **4**=*S. gyalocarpa*; **5**=*S. leucoblepharis*; **6**=*S. parahumboldtii*; **7=***S. impressula*; **8**=*S. ocaniesnsis*; **9**=*S. speudosylvatica*; **10**= *S. luteocyphelata*; **11**= *S. leneariloba*, 12=*S. caulescens*.

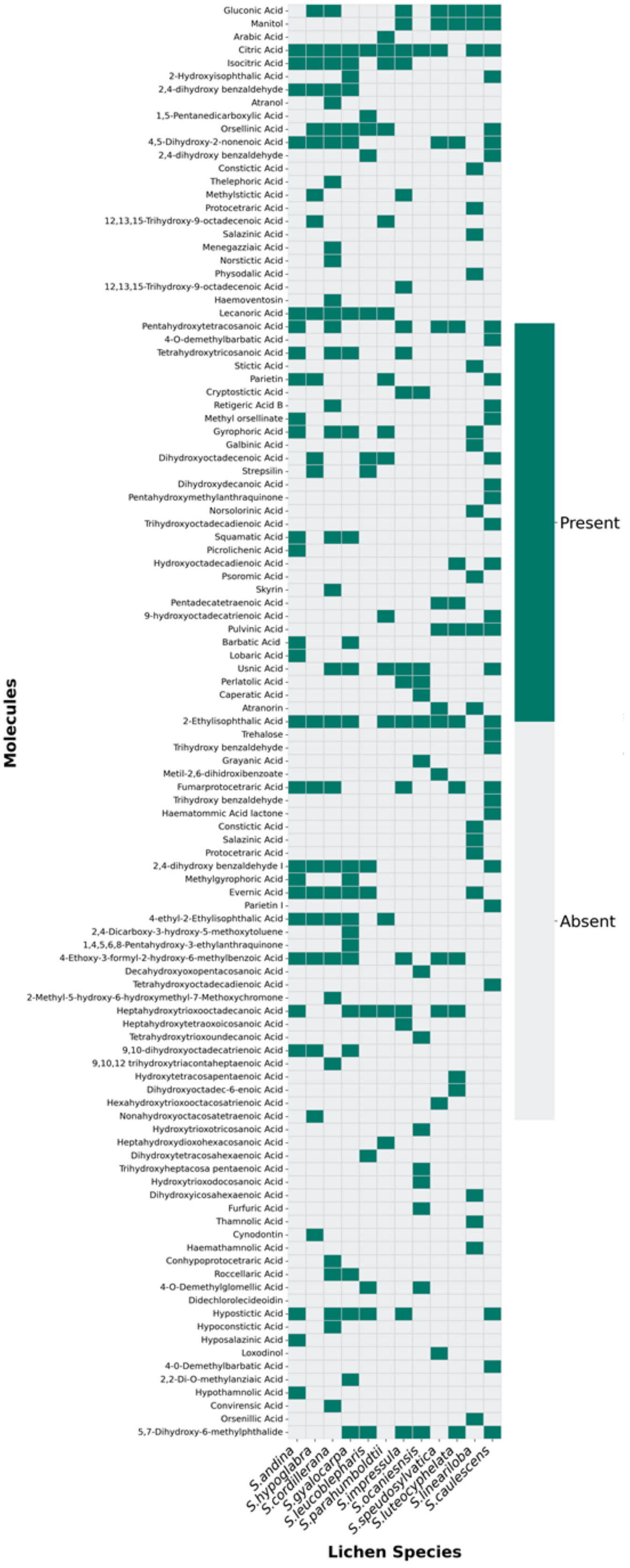

Figure S1. The absence and presence of molecules in the 12 different species of Sticta lichens is shown. These analyses are derived from the digital database, where their SMILES and the species to which they belong can be found.

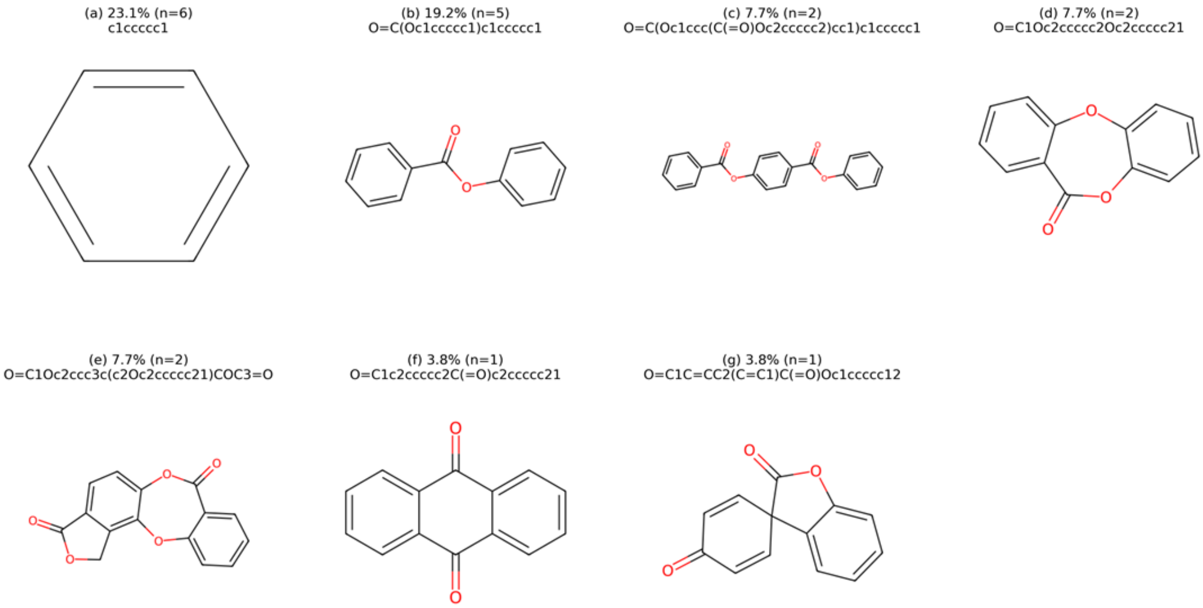

Figure S2. Predominant ring-containing scaffolds identified in the S. andina. The frequency of each scaffold (as a percentage).

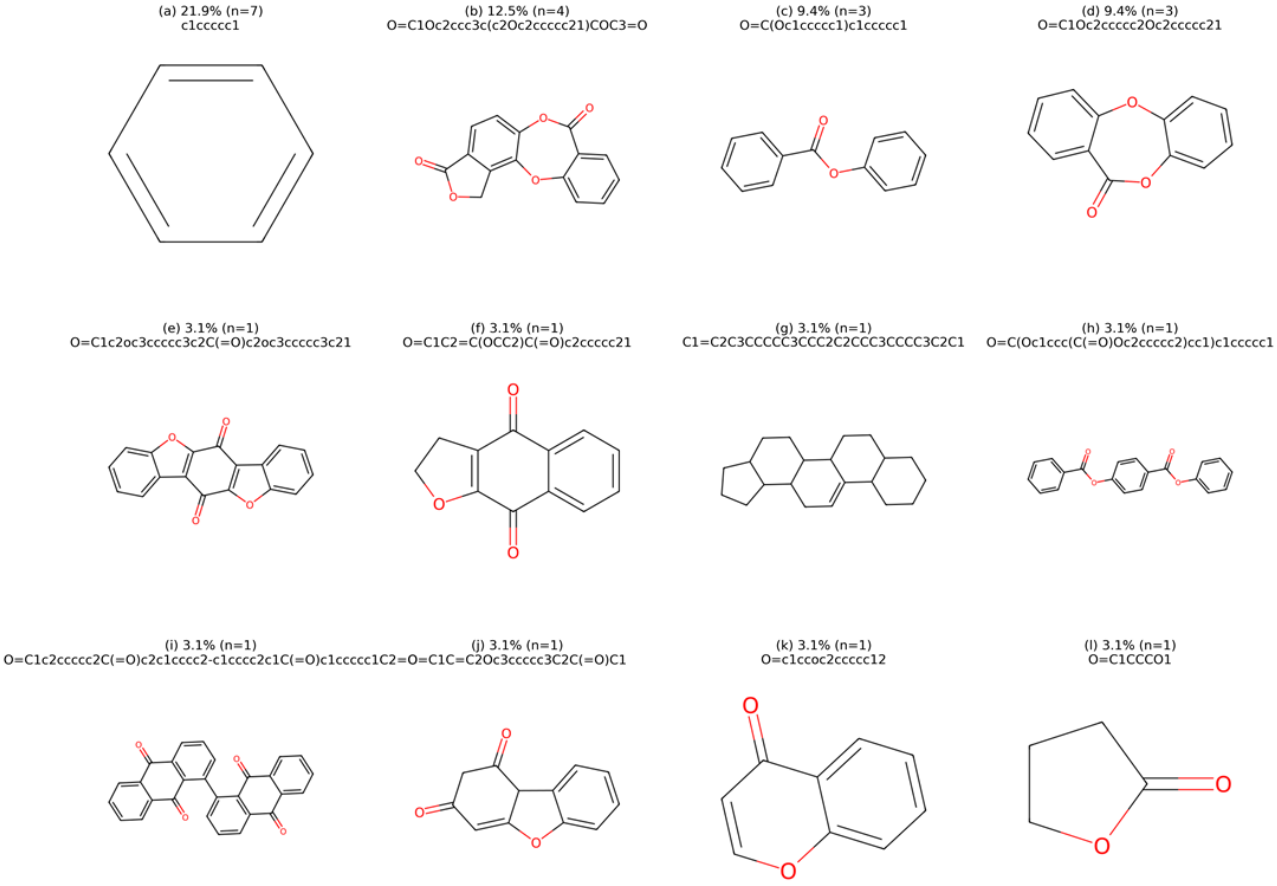

Figure S3. Predominant ring-containing scaffolds identified in the *S. cordillerana*. The frequency of each scaffold (as a percentage).

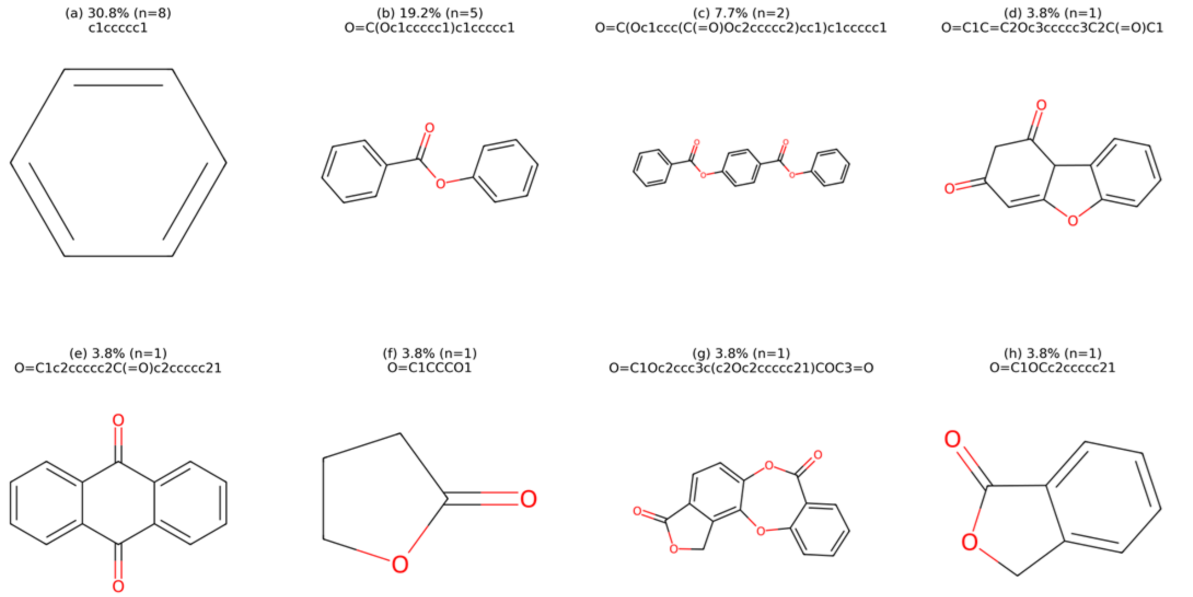

Figure S4. Predominant ring-containing scaffolds identified in the *S. gyalocarpa*. The frequency of each scaffold (as a percentage).

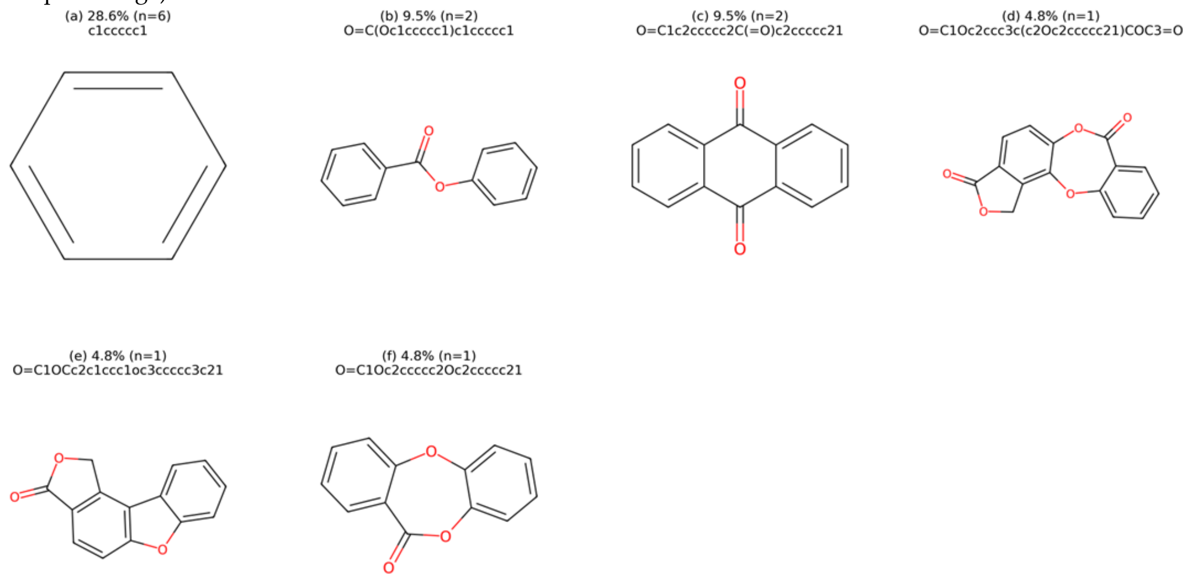

Figure S5. Predominant ring-containing scaffolds identified in the S. hypoglabra. The frequency of each scaffold (as a percentage).

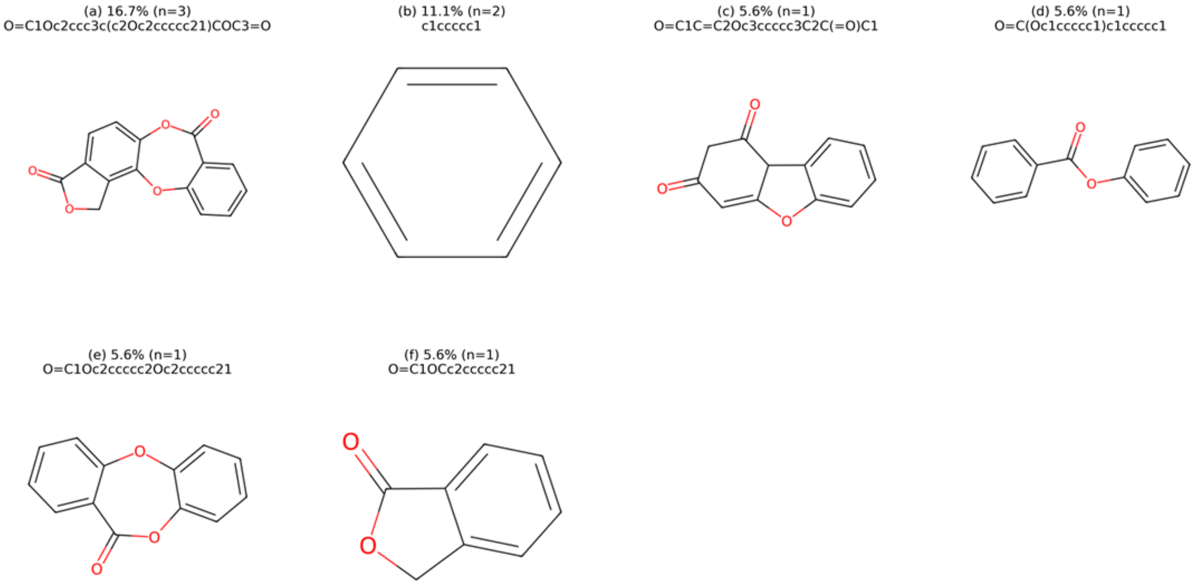

Figure S6. Predominant ring-containing scaffolds identified in the S. impressula. The frequency of each scaffold (as a percentage).

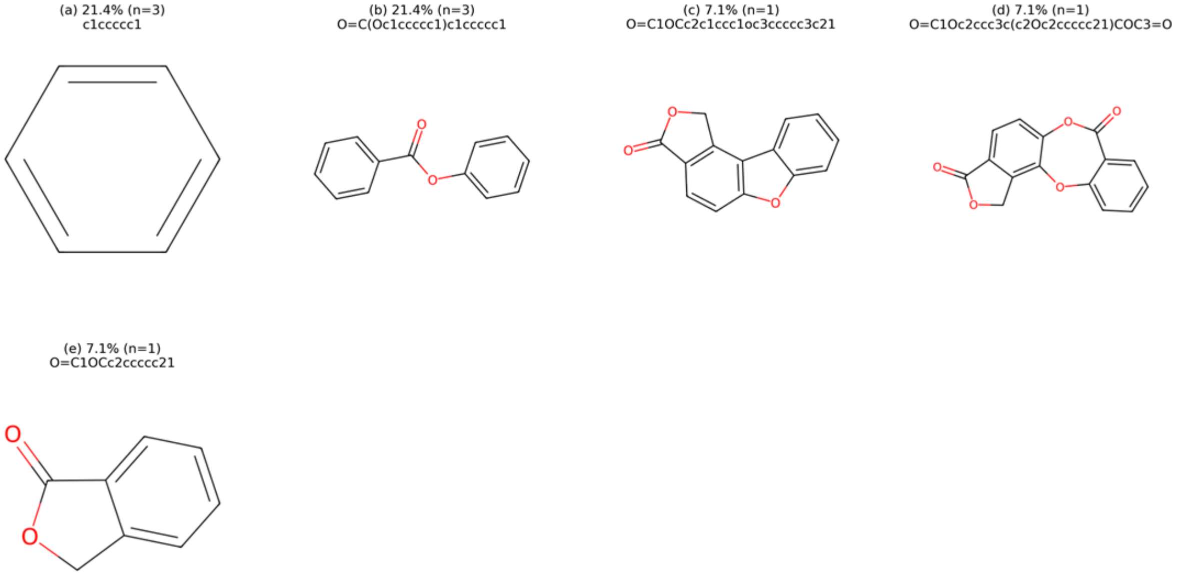

Figure S7. Predominant ring-containing scaffolds identified in the *S. leucoblepharis*. The frequency of each scaffold (as a percentage).

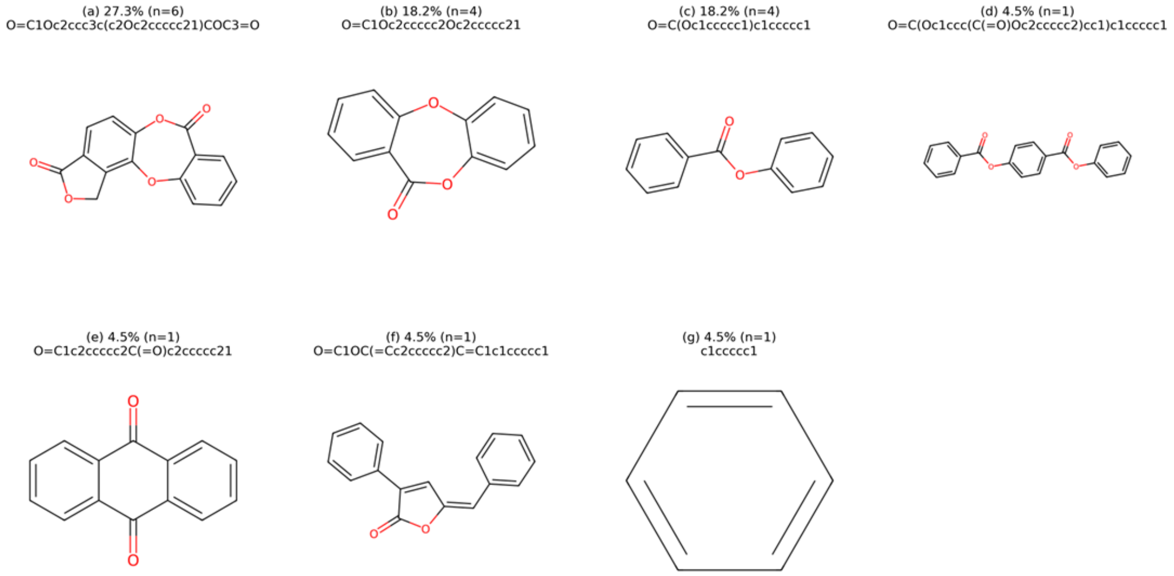

Figure S8. Predominant ring-containing scaffolds identified in the *S. lineariloba*. The frequency of each scaffold (as a percentage).

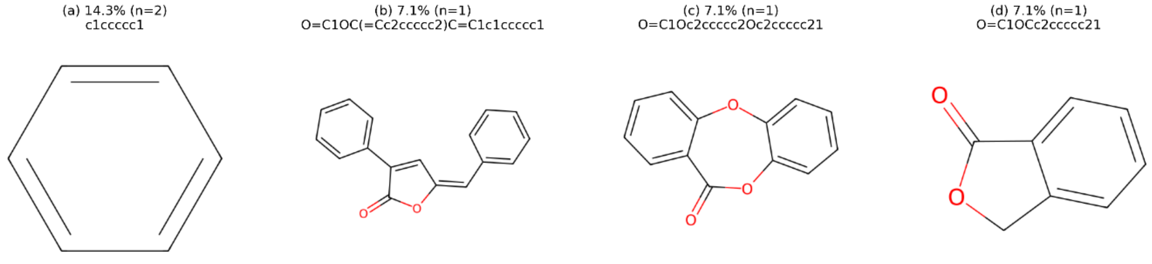

Figure S9. Predominant ring-containing scaffolds identified in the *S. luteocyphelata*. The frequency of each scaffold (as a percentage).

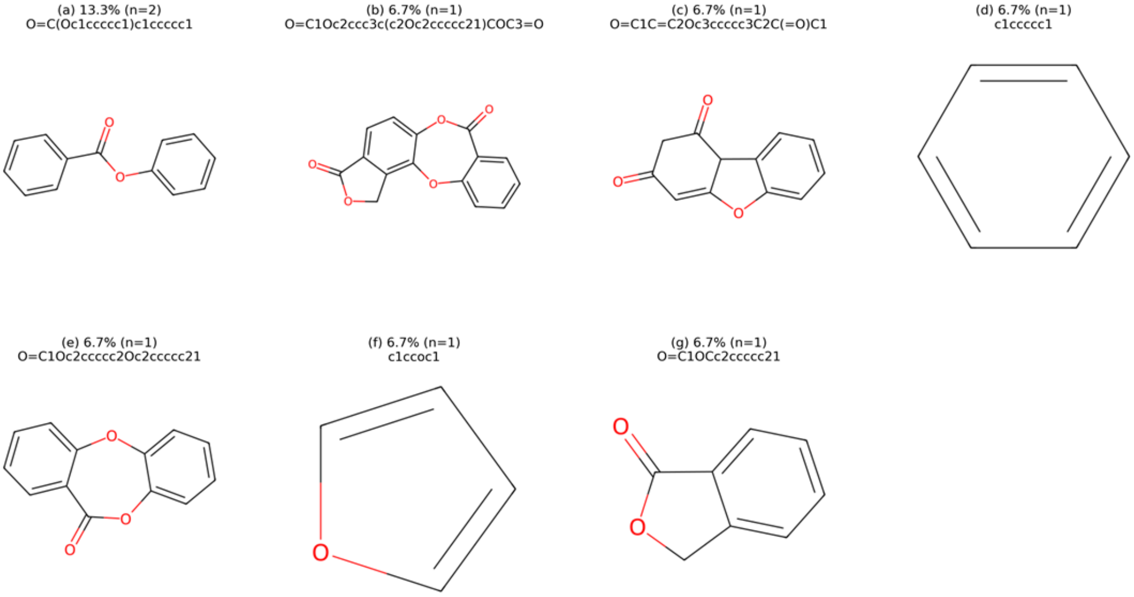

Figure S10. Predominant ring-containing scaffolds identified in the *S. ocaniesnsis*. The frequency of each scaffold (as a percentage).

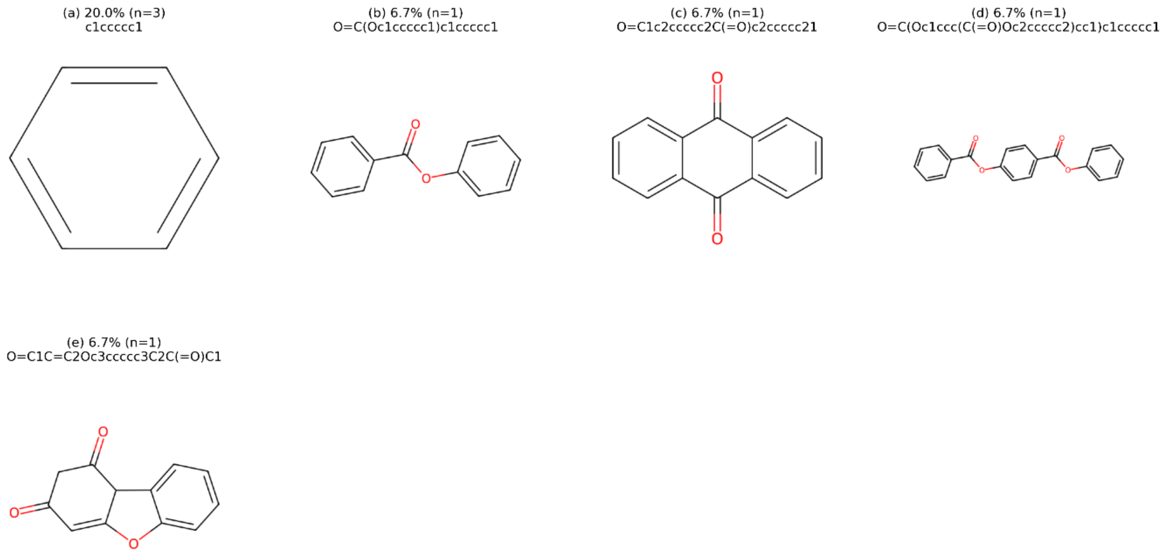

Figure S11. Predominant ring-containing scaffolds identified in the *S. parahumboldtii*. The frequency of each scaffold (as a percentage).

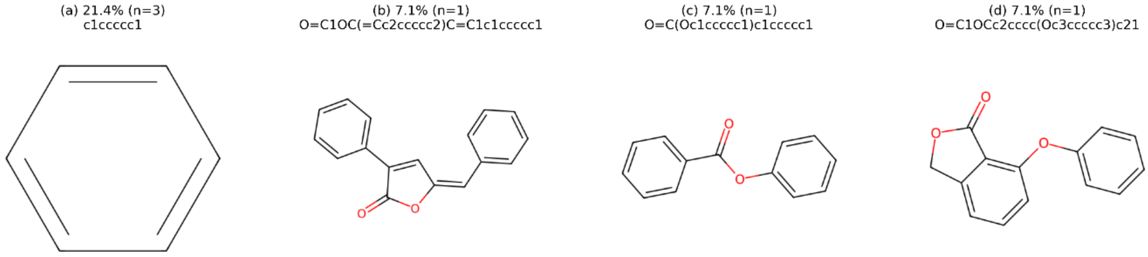

Figure S12. Predominant ring-containing scaffolds identified in the *S. speudosylvatica*. The frequency of each scaffold (as a percentage).
